# Uniform sarcolemmal dystrophin expression is required to prevent extracellular microRNA release and improve dystrophic pathology

**DOI:** 10.1002/jcsm.12506

**Published:** 2019-12-17

**Authors:** Tirsa L.E. van Westering, Yulia Lomonosova, Anna M.L. Coenen‐Stass, Corinne A. Betts, Amarjit Bhomra, Margriet Hulsker, Lucy E. Clark, Graham McClorey, Annemieke Aartsma‐Rus, Maaike van Putten, Matthew J.A. Wood, Thomas C. Roberts

**Affiliations:** ^1^ Department of Physiology, Anatomy and Genetics University of Oxford South Parks Road Oxford UK; ^2^ Department of Paediatrics University of Oxford South Parks Road Oxford UK; ^3^ Department of Human Genetics Leiden University Medical Center Leiden the Netherlands; ^4^ Sanford Burnham Prebys Medical Discovery Institute, Development, Aging and Regeneration Program La Jolla CA USA

**Keywords:** Duchenne muscular dystrophy, MicroRNA, Biomarkers, Dystrophin, X‐chromosome inactivation, Muscle turnover

## Abstract

**Background:**

Duchenne muscular dystrophy (DMD) is a fatal muscle‐wasting disorder caused by genetic loss of dystrophin protein. Extracellular microRNAs (ex‐miRNAs) are putative, minimally invasive biomarkers of DMD. Specific ex‐miRNAs (e.g. miR‐1, miR‐133a, miR‐206, and miR‐483) are highly up‐regulated in the serum of DMD patients and dystrophic animal models and are restored to wild‐type levels following exon skipping‐mediated dystrophin rescue in *mdx* mice. As such, ex‐miRNAs are promising pharmacodynamic biomarkers of exon skipping efficacy. Here, we aimed to determine the degree to which ex‐miRNA levels reflect the underlying level of dystrophin protein expression in dystrophic muscle.

**Methods:**

Candidate ex‐miRNA biomarker levels were investigated in *mdx* mice in which dystrophin was restored with peptide‐PMO (PPMO) exon skipping conjugates and in *mdx‐Xist* ^Δhs^ mice that express variable amounts of dystrophin from birth as a consequence of skewed X‐chromosome inactivation. miRNA profiling was performed in *mdx‐Xist* ^Δhs^ mice using the FirePlex methodology and key results validated by small RNA TaqMan RT‐qPCR. The muscles from each animal model were further characterized by dystrophin western blot and immunofluorescence staining.

**Results:**

The restoration of ex‐myomiR abundance observed following PPMO treatment was not recapitulated in the high dystrophin‐expressing *mdx‐Xist* ^Δhs^ group, despite these animals expressing similar amounts of total dystrophin protein (~37% of wild‐type levels). Instead, ex‐miRNAs were present at high levels in *mdx‐Xist* ^Δhs^ mice regardless of dystrophin expression. PPMO‐treated muscles exhibited a uniform pattern of dystrophin localization and were devoid of regenerating fibres, whereas *mdx‐Xist* ^Δhs^ muscles showed non‐homogeneous dystrophin staining and sporadic regenerating foci.

**Conclusions:**

Uniform dystrophin expression is required to prevent ex‐miRNA release, stabilize myofiber turnover, and attenuate pathology in dystrophic muscle.

## 1 Introduction

Duchenne muscular dystrophy (DMD) is a classical monogenic disorder characterized by severe muscle weakness. Pathology typically manifests at ~3 years of age, with the majority of patients losing ambulation around the ages of 10–12. Progressive muscle loss ultimately culminates in fatality due to failure of the heart and diaphragm muscles, typically in the 2nd–4th decade of life. The genetic cause of DMD is disruption of the gene encoding the dystrophin protein (DMD) located on the X‐chromosome (Xp21). DMD is thus an X‐linked recessive disorder that almost exclusively affects male patients. Dystrophin is a structural and signaling protein that is localized at the sarcolemma and acts as an organizing centre for the dystrophin‐associated protein complex.^1^ As such, dystrophin forms a mechanical link between the actin cytoskeleton and the extracellular matrix via the dystrophin‐associated protein complex^2^ and functions as a ‘shock absorber’ that protects myofibers from contractile damage.^3^

Female carriers of DMD‐causing mutations do not generally present with muscle pathology on account of the presence of a functional copy of the DMD gene on the alternate X‐chromosome. However, female carriers typically express >60% of normal dystrophin protein^4^, ^5^ as a consequence of random epigenetic silencing of the ‘healthy’ chromosome via X‐chromosome inactivation (XCI). Dystrophin positive myofibers in female carriers experience a selection advantage and become progressively enriched with age, as they are resistant to contraction‐induced damage.^4^ However, 3–8% of female carriers present with myopathic symptoms with a range of severities and variable cardiac involvement.^6^ While the reasons for disease manifestation in carriers are not well understood, it has been suggested that skewed XCI results in reduced levels of dystrophin synthesis from the healthy allele.^4^, ^7^ Conversely, Brioschi *et al*. found that skewed XCI was not clearly associated with dystrophic pathology, suggesting that the situation is likely more complex.^8^

At present, there is no cure for DMD. Almost all patients receive corticosteroid therapy (e.g. prednisone or deflazacort) that has been shown to moderately delay disease progression and prolong ambulation.^9^ However, steroid therapy does not treat the underlying cause of the disease and is associated with side effects including excessive weight gain, Cushingoid symptoms, and changes in behaviour. Much research effort has been directed towards the development of interventions that can prolong survival and improve quality of life in DMD patients. One of the most promising molecular therapy approaches is antisense oligonucleotide‐mediated exon skipping, in which splice modulation is utilized to restore the dystrophin translation reading frame.^10^ However, restoration of dystrophin protein in patient muscles remains a significant technical challenge. This is exemplified by the controversy surrounding the approval of the exon skipping compound eteplirsen by the US FDA,^11^ given that this drug was shown to achieve a mean restoration of less than 1% of normal dystrophin protein expression.^12^ The minimum amount of dystrophin required for therapeutic benefit is therefore a question of key importance. Becker muscular dystrophy (BMD), a related dystrophinopathy, is characterized by a later onset and slower disease progression and is principally caused by in‐frame dystrophin mutations. Dystrophin protein expression levels vary widely between patients, but high levels of dystrophin are frequently associated with less severe disease manifestation,^13^ although the situation is complicated when considering the location of the dystrophin mutation within the DMD gene.^14^ Similarly, expression of ~30% of a near‐normal dystrophin protein in the muscles of X‐linked dilated cardiomyopathy patients is sufficient to prevent skeletal muscle dystrophic pathology (although the total absence of dystrophin in the heart still leads to cardiac pathology).^15^

Heterozygous *mdx* mice (expressing dystrophin in the heart at the level of ~50%) experience only mild cardiomyopathy,^16^ and cardiac muscle exhibits minor functional improvements with even as little as ~3% dystrophin expression.^17^ Further studies from our group suggest that therapeutic re‐introduction of ~15% of normal dystrophin in adult *mdx* mice is sufficient to provide protection against contractile damage.^18^

Importantly, the development of experimental therapies for DMD has advanced at a faster pace than that of biochemical endpoints, and there is consequently an unmet need for minimally invasive pharmacodynamic biomarkers for DMD and dystrophin protein re‐expression.^19^, ^20^, ^21^ One class of potential molecular biomarkers is the microRNAs (miRNAs).^21^ These short RNA molecules (~22 nucleotides) are involved in the control of gene regulation in cells during a variety of physiological and pathophysiological processes (including myogenic differentiation, fibrosis, and inflammation).^22^, ^23^, ^24^ miRNAs are abundant and stable in biofluids,^25^ and muscle‐enriched miRNAs (the myomiRs: miR‐1, miR‐133, and miR‐206) have been shown to be highly elevated in the serum of DMD patients and dystrophic animal models.^26^, ^27^, ^28^, ^29^ Furthermore, we have previously suggested that circulating myomiR levels reflect the degree of turnover in dystrophic muscle^30^ and that regenerating fibers substantially contribute to extracellular miRNA (ex‐miRNA) release.^31^

Circulating myomiRs are restored towards wild‐type levels after a single intravenous dose of a peptide‐phosphorodiamidate morpholino oligomer (PPMO) conjugate designed to rescue dystrophin expression by exon skipping in the *mdx* mouse,^28^ and similar results have also been reported with expressed U1/U7 snRNA‐based exon skipping systems.^26^, ^32^ Importantly, we observed that restoration of ex‐miRNA levels was commensurate with the degree of dystrophin restoration when comparing two PPMO conjugates of different potencies, suggesting that these molecules might constitute pharmacodynamic biomarkers of dystrophin restoration in DMD patients.^30^ However, the degree to which ex‐miRNA (and other) biomarkers reflect the underlying level of dystrophin protein expression in muscle remains an unresolved question. We therefore aimed to investigate the relationship between dystrophin protein expression and the abundance levels of circulating miRNAs. Interestingly, we found that a uniform pattern of dystrophin protein distribution is required for therapeutic restoration of ex‐miRNA levels and, by extension, stabilization of myofiber turnover in dystrophic muscle.

## 2 Methods

### 2.1 Animal studies

Animals were housed at the Biomedical Sciences Unit (University of Oxford UK) or LUMC (Leiden University Medical Center, the Netherlands) under 12:12 h light:dark conditions with access to food and water *ad libitum*. Experiments performed in the UK were authorized and approved by the University of Oxford ethics committee and UK Home Office (project license 30/2907). Experiments performed in the Netherlands were authorized and approved by the Animal Experimental Commission (DEC) of the LUMC and conform with Directive 2010/63/EU of the European Parliament.

Heterozygote carriers (*n* = 4) were generated by crossing male C57BL/10ScSn (C57) mice with female *mdx* (C57BL/10ScSn‐*Dmd* ^*mdx*^/J) mice. The resulting female progeny was sacrificed at 8 weeks of age by escalating CO_2_ concentration, and serum and tissues were collected (*n* = 4). Serum and tissue from age‐matched and sex‐matched C57 (*n* = 4) and homozygous *mdx* (*n* = 4) were collected in parallel.

*mdx‐Xist* ^Δhs^ mice were generated by crossing male *mdx* mice with female *Xist* ^Δhs^ mice as described previously.^33^ Non‐terminal tail vein serum collections were performed on female *mdx‐Xist* ^Δhs^ and sex‐matched *mdx*, C57 and *Xist* ^Δhs^ at 4, 8, 12, 16, 20, 24, 28, 32, 40, and 48 weeks of age (*n* = 5 per group). Animals were sacrificed by cervical dislocation at 78 weeks of age, and tissues collected.

Male and female C57 and *mdx* mice (*n* = 4 for each group) were sacrificed at 10, 24, 52, and 80 weeks of age by increasing CO_2_ concentration, and serum and tissues collected.

### 2.2 Serum collection

Blood was harvested from the jugular vein or via an angled tail vein cut and collected in Microvette CB 300 collection tubes (Sarstedt, Nümbrecht, Germany). Blood was incubated at 4°C for 2–4 h to allow for clotting, after which samples were centrifuged at 10,000 g for 5 min, and the supernatant transferred to a fresh tube. Serum samples were stored at −80°C until ready for use.

### 2.3 Antisense oligonucleotide‐mediated exon skipping

The cell penetrating peptide Pip9b2 (RXRRBRRFQILYRBRXRB, where X = aminohexanoic acid, and B = β‐alanine) was conjugated to a PMO (5′‐GGCCAAACCTCGGCTTACCTGAAAT‐3′; Gene Tools LLC, Philomath, OR, USA) through an amide linkage between the C‐terminus of the peptide and the amine of the morpholine heterocycle at the 3′ terminus of the PMO.^34^ Conjugates were subsequently dissolved in sterile water and filtered through a 0.22 μm cellulose acetate membrane. Male *mdx* mice received a single intravenous injection of 12.5 mg/kg Pip9b2‐PMO conjugate dissolved in 0.9% saline at 6 weeks of age (*n* = 4). Two weeks after administration, Pip9b2‐PMO‐treated mice and their age‐matched and sex‐matched controls (C57 *n* = 4, and *mdx*, *n* = 3) were sacrificed, and serum and tissues collected at 8 weeks of age.

### 2.4 FirePlex serum miRNA profiling

FirePlex analysis was performed as a service by AbCam (Cambridge, UK). Briefly, miRNA‐specific polyethylene glycol‐based hydrogel particles were used to capture target miRNAs. These miRNAs were subsequently amplified by PCR, eluted, re‐amplified in a second PCR reaction, re‐hybridized to the probe particles, incubated with a fluorescent reporter, and the fluorescence of each particle determined by flow cytometry. For the FirePlex profiling analyses, miRNAs were measured directly from serum, and data were obtained in relative fluorescence units. All target miRNAs were detected at levels above background. Artificial calibrator bridge samples (consisting of a mixture of C57 and *mdx* serum samples) were included in each FirePlex run to enable measurements from two FirePlex experiments to be combined.

Raw probe signals were background subtracted and normalized to a synthetic ‘normalizer probe’. Normalizer probes were generated by first calculating the geometric mean of miR‐16‐5p, miR‐17‐5p, and miR‐92a‐3p for each sample and then dividing all means by the geometric average of all samples. As such, the resultant normalizer probes are “deflated” (i.e. such that the average of all deflated normalizer probe values is equal to one). Probe signal intensities were then divided by the deflated normalizer probe in order to normalize the data while preserving the natural range of fluorescence intensity values. For the heatmap and principal component analysis (PCA), data were log_2_ transformed. All other analyses utilized data whereby abundance ratios were scaled such that the mean of the C57 control group was returned to a value of 1. Normalized and raw signal intensities are provided in Supplementary File S1.

### 2.5 Western blot

Protein was extracted using RIPA lysis and extraction buffer (ThermoFisher Scientific, Abingdon, UK), supplemented with 5% SDS. Samples were mixed with NuPAGE sample reducing agent and NuPAGE LDS sample buffer (both ThermoFisher Scientific) and then size separated on a Tris‐Acetate 3–8% gradient gel (ThermoFisher Scientific). Protein was transferred to a 0.45 μm polyvinylidene fluoride membrane and incubated in Odyssey blocking buffer (LI‐COR Biosciences Ltd, Cambridge, UK) for 1 h at room temperature. Membranes were subsequently probed with anti‐dystrophin (1:100 dilution; NCL‐DYS1, Leica Microsystems (UK) Ltd, Milton Keynes, UK) and anti‐vinculin antibodies (1:100,000 dilution; V9131, Sigma Aldrich, Gillingham, UK) overnight at 4°C. Membranes were incubated with goat anti‐mouse secondary antibody (P/N 925‐32210, LI‐COR Biotechnology) for 1 h at room temperature and imaged using Odyssey Fc Imager (LI‐COR Biotechnology).

### 2.6 RNA extraction

Total RNA from serum (either 25 μl or 50 μl) was extracted using TRIzol LS Reagent (ThermoFisher Scientific) according to manufacturer's instructions. An exogenous control synthetic oligonucleotide (3 μl of 5 nM cel‐miR‐39; 5′‐UCACCGGGUGUAAAUCAGCUUG‐3′) was added at the phenolic extraction step to act as exogenous reference control for RT‐qPCR analysis. RNA from tissue cryosections was extracted using TRIzol Reagent (ThermoFisher Scientific) according to manufacturer's instructions.

### 2.7 miRNA RT‐qPCR

miRNA quantification was performed by small RNA TaqMan RT‐qPCR as described previously.^35^, ^36^ Briefly, RNA was reverse transcribed using the TaqMan MicroRNA Reverse Transcription Kit (ThermoFisher Scientific) according to manufacturer's instructions. cDNA was amplified using a StepOne Plus real‐time PCR Thermocycler (ThermoFisher Scientific) with TaqMan Gene Expression Master Mix (ThermoFisher Scientific) using universal cycling conditions; 95°C for 10 minutes, followed by 45 cycles of 95°C for 15 seconds, 60°C for 1 minute. All reactions were performed in duplicate. RT‐qPCR data was analysed using the Pfaffl method^37^ and PCR efficiencies determined by amplification curve analysis using LinRegPCR.^38^ ex‐miRNA qPCR assays were normalized to the exogenous cel‐miR‐39 spike‐in oligonucleotide. miRNA primer/probe mixes (ThermoFisher Scientific) used in this study are listed in **Table** **S1**.

### 2.8 Exon skipping RT‐qPCR

Complementary DNA was generated from 500 ng of total RNA using the High‐Capacity cDNA Reverse Transcription Kit (ThermoFisher Scientific) according to manufacturer's instructions. The resulting cDNA was diluted 1:5 prior to analysis. cDNA was amplified using a StepOne Plus real‐time PCR Thermocycler (ThermoFisher Scientific) with TaqMan Gene Expression Master Mix (ThermoFisher Scientific) using universal cycling conditions: 95°C for 10 min, followed by 45 cycles of 95°C for 15 s, 60°C for 1 min. All reactions were performed in duplicate. The percentage of DMD exon 23 skipping was determined by calculating [1–the ratio of unskipped (exons 23–24):total (exons 20–21) DMD transcripts] × 100%. Sequences of primers/probes utilized are listed in Table S2.

### 2.9 Immunofluorescence

Tibialis anterior (TA) muscles were mounted onto corks using Tissue‐TEK O.C.T. Compound (VWR, Lutterworth, UK). Frozen muscles were cryosectioned (8 μm) in either transverse or longitudinal orientation and stored at −80°C. Slides were subsequently air‐dried and soaked in phosphate buffered saline (PBS). For dystrophin staining, sections were blocked in PBS supplemented with 20% normal goat serum and 20% foetal bovine serum for 2 h at room temperature. Slides were then incubated with anti‐dystrophin (1:1,000 dilution, ab15277, AbCam), anti‐LAMA2 (laminin α2) (1:1,000 dilution, L0663, Sigma Aldrich), anti‐dystrobrevin alpha (1:100, in‐house antibody; α1CTFP)^39^, and anti‐nitric oxide synthase 1 (1:100, ab76067, AbCam) antibodies for 2 h at room temperature (anti‐dystrobrevin alpha and anti‐nitric oxide synthase 1 antibodies were a kind gift from Ben Edwards and Prof. Kay Davies, University of Oxford). After washing, sections were incubated with fluorescent secondary antibodies for 1 h at room temperature; goat anti‐rabbit and goat anti‐rat (1:500 dilution, AlexaFluor 595 and AlexaFluor 488, respectively, ThermoFisher Scientific). For embryonic myosin heavy chain (eMHC) staining, muscle sections were incubated in acetone for 10 min at −20°C, air‐dried at room temperature, and blocked in 20% normal goat serum in PBS with 0.1% Triton X‐100 for 1 h. Sections were incubated with anti‐MYH3 (eMHC) (1:100 dilution, F1.652‐AlexaFluor 594, Santa Cruz Biotechnology, Dallas, TX, USA) and monoclonal anti‐LAMA2 (as above) in blocking solution for 2 h at room temperature. After washing with PBS, sections were incubated for 1 h with AlexaFluor 488‐coupled goat‐anti‐rat secondary antibodies (1:500 dilution, ThermoFisher). Sections were incubated with DAPI (1:5,000 dilution, ThermoFisher) for 15 min during washings after the secondary antibodies at room temperature. Slides were mounted in Vectashield Hard Set mounting medium (Vector Laboratories, Peterborough, UK). Myofiber cross‐sectional area was measured using a custom ImageJ macro. For quantification of eMHC positivity, centrally nucleated fibers and myofiber cross‐sectional area multiple images were acquired and at least 419 fibers counted per animal (range 419 to 1,868 myofibers).

### 2.10 Statistical analysis and bioinformatics

FirePlex data were processed and normalized using the Firefly analysis workbench v2.0.91 (AbCam). Multiple experiment viewer (MeV) (The Institute for Genomic Research, Rockville, MD, USA)^40^ was used to perform Student's *t*‐test and one‐way ANOVA analyses, and for heatmap visualization. PCA was performed in R using the *prcomp* function. The following additional analyses were performed in graphpad prism 7 (GraphPad Software Inc, La Jolla, CA); Student's *t*‐test, Shapiro‐Wilk test for normality, one‐way ANOVA with Tukey *post hoc* test, Kruskal‐Wallis one‐way ANOVA with Dunn's *post hoc* test, Pearson/Spearman correlation analysis, receiver operating characteristic (ROC) analysis, and cumulative distribution function (CDF) analysis. Where experiments had large samples sizes (i.e. *n* > 10), normality was tested, and parametric and non‐parametric tests of significance/correlation applied as appropriate.

## 3 Results

### 3.1 Extracellular myomiRs are not elevated in heterozygous *mdx* mice

**Figure 1:**
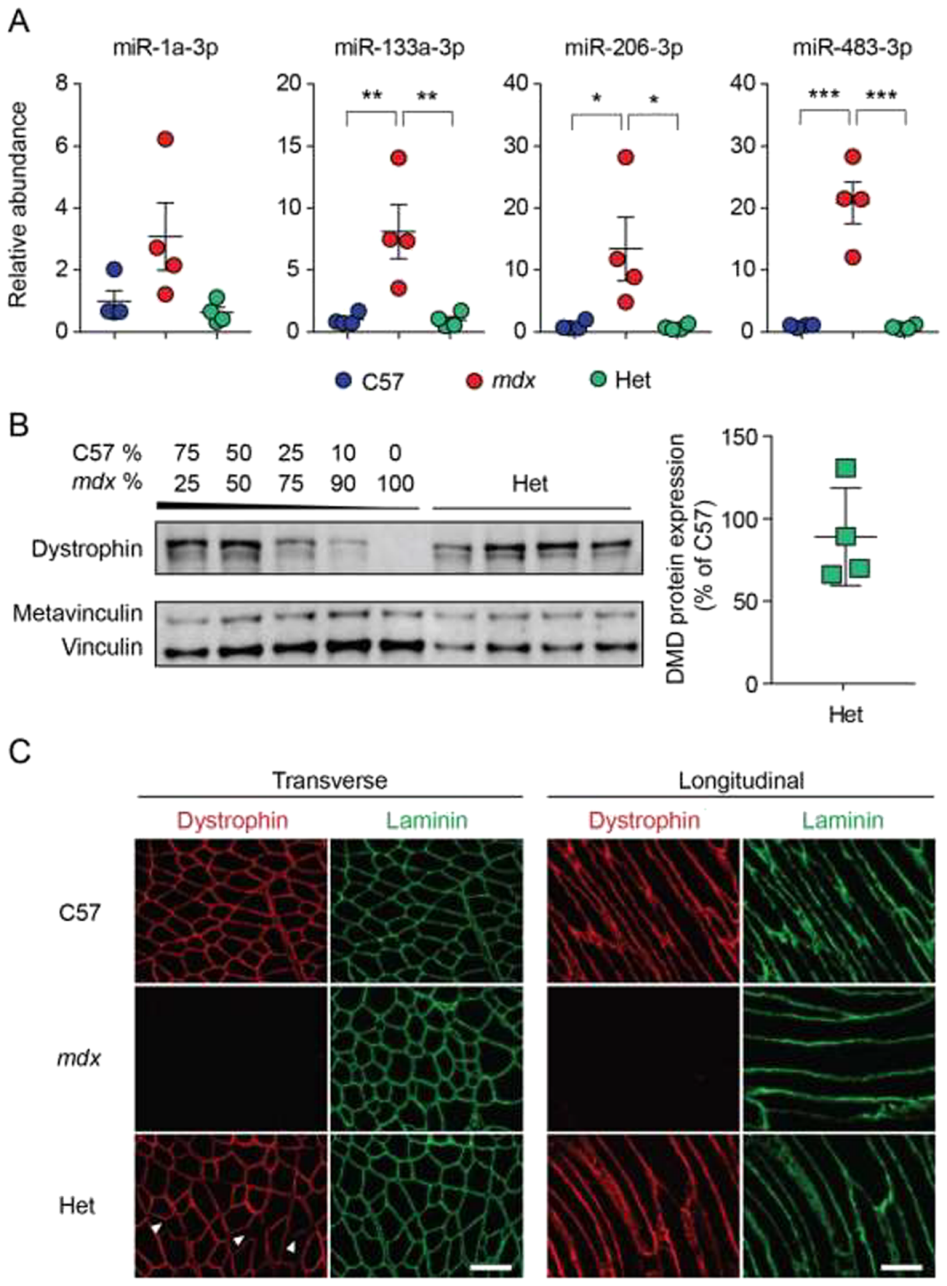
Analysis of serum miRNA levels and dystrophin expression in *mdx* heterozygote animals. (*A*) ex‐miRNA abundance in serum of C57, *mdx*, and *mdx* heterozygote (Het) animals (all 8‐week‐old female animals, *n* = 4) was determined by small RNA TaqMan RT‐qPCR and normalized to a synthetic spike‐in control oligonucleotide (cel‐miR‐39). Abundance ratios were scaled such that the mean of the C57 control was returned to a value of 1. (*B*) Western blot analysis of dystrophin protein expression in tibialis anterior muscle from C57, *mdx*, and *mdx* heterozygotes. Vinculin was utilized as a loading control. A standard curve, consisting of mixtures of C57 and *mdx* protein lysates in ratios as indicated, was used to quantify dystrophin protein expression in the *mdx* heterozygotes. (*C*) Representative immunofluorescence staining of dystrophin protein in transverse and longitudinal tibialis anterior sections for C57, *mdx*, and *mdx* heterozygote animals. Sections were co‐stained with laminin to label myofibers. Arrowheads indicate myofibers with weaker dystrophin staining. All values are mean ± standard error of the mean, *P < 0.05, **P < 0.01, ***P < 0.001, one‐way analysis of variance and Tukey *post hoc* test. Scale bars represent 100 μm, images taken at ×20 magnification.

It has previously been reported that the myomiR miR‐206‐3p is elevated in human female DMD carriers.^41^ We therefore decided to first investigate ex‐miRNA abundance in heterozygous female carrier mice. Serum was harvested from 8‐week‐old female C57 (wild‐type), *mdx* homozygous (dystrophin‐deficient), and *mdx* heterozygous mice (*n* = 4), and the levels of ex‐miRNAs (miR‐1a‐3p, miR‐133a‐3p, miR‐206‐3p, and miR‐483‐3p) were determined by RT‐qPCR. The myomiRs are the most widely reported miRNAs to be elevated in dystrophic serum,^21^ whereas we recently identified miR‐483‐3p as a promising new candidate DMD biomarker.^29^ All miRNAs were up‐regulated in the female *mdx* mice and reached statistical significance at the *P* < 0.05 level in the cases of miR‐133a‐3p, miR‐206‐3p, and miR‐483‐3p (*Figure* 1A). However, there was no difference between C57 and *mdx* heterozygous mice for all miRNAs tested. Western blot analysis of TA muscles revealed that dystrophin was expressed at an average of 89% of wild‐type levels in the *mdx* heterozygotes (*Figure* 1B). Dystrophin staining in transverse and longitudinal TA sections showed that while *mdx* homozygotes were almost completely devoid of dystrophin‐positive fibers, *mdx* heterozygote myofibers were almost entirely dystrophin positive (*Figure* 1C). Notably, the intensity of the dystrophin staining was slightly less uniform than for the C57 controls (see arrowheads in *Figure* 1C). These data show that dystrophin expression levels are higher than 50% in *mdx* heterozygotes at this age, likely as a consequence of the gradual replacement of dystrophin‐negative degenerating fibers with dystrophin‐positive fibers during regeneration.^42^, ^43^, ^44^

### 3.2 Dystrophic mice with skewed X‐chromosome inactivation express varying levels of dystrophin protein

**Figure 2:**
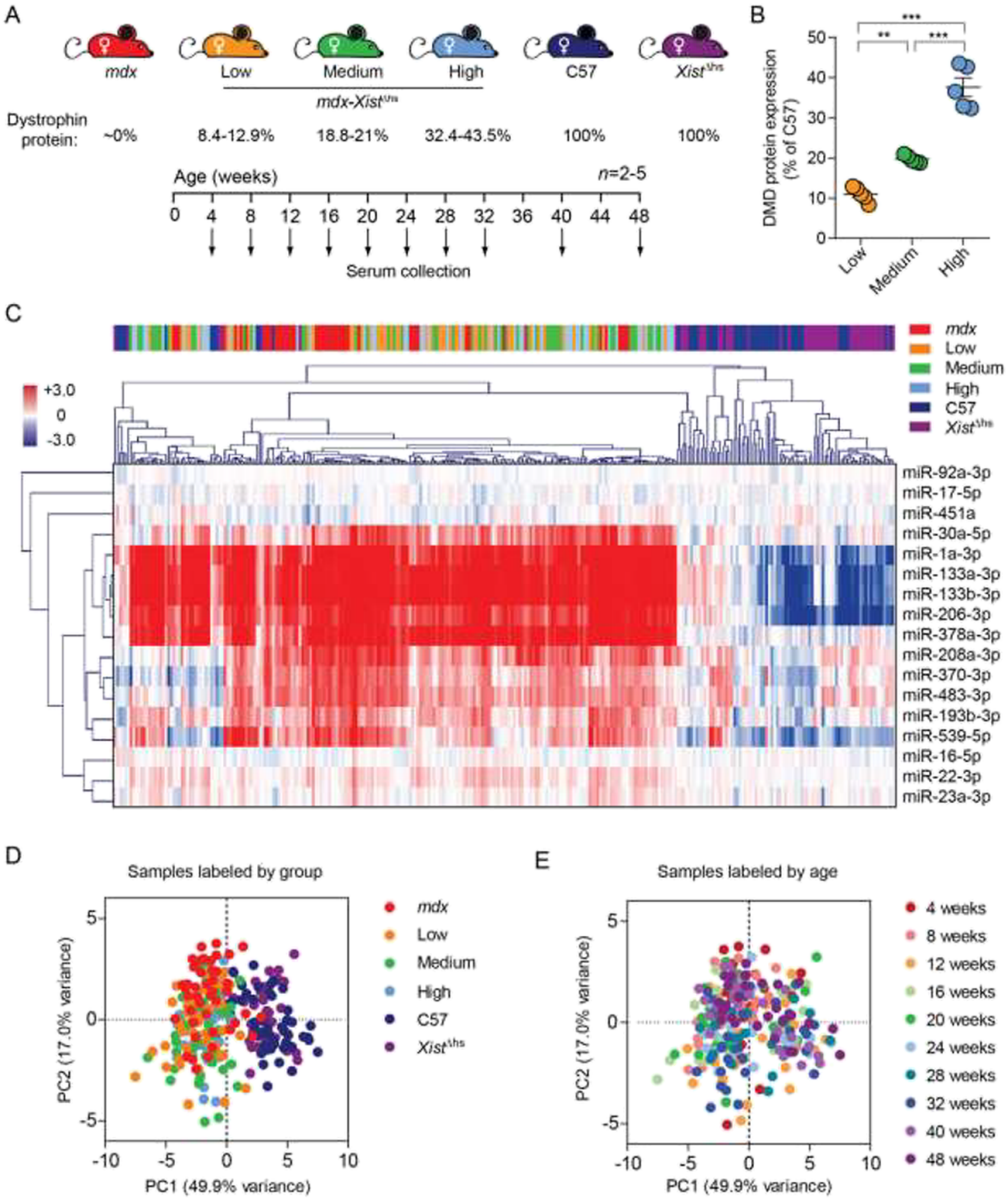
Longitudinal analysis of serum miRNA levels in mice expressing various levels of dystrophin protein. (*A*) Experimental design of longitudinal miRNA profiling study. Male *mdx* and female *Xist* ^Δhs^ mice were bred, and the female mice from the F1 generation (*mdx‐Xist* ^Δhs^) were assigned to low, medium, or high dystrophin expression groups based on dystrophin Western blot analysis of quadriceps femoris muscle lysates. Serum for miRNA analysis was collected via the tail vein for all *mdx‐Xist* ^Δhs^ mice at the ages indicated (4–48 weeks of age). Additionally, wild‐type (C57 and *Xist* ^Δhs^) and dystrophic (*mdx*) control serum samples were collected in parallel. All samples were female mice, *n* = 5 measurements for each group at each age in the majority of cases (range: 4–5, except for *mdx* 28 weeks *n* = 2). (*B*) Quantification of dystrophin protein in all *mdx‐Xist* ^Δhs^ mice. Values are mean ± standard error of the mean, **P < 0.01, ***P < 0.001, one‐way analysis of variance and Tukey *post hoc* test. Serum levels were determined for a custom panel of 17 miRNAs using the FirePlex method, and miRNA data normalized to the geometric average of three stable reference miRNAs: miR‐16‐5p, miR‐17‐5p, and miR‐92a‐3p. (*C*) Heatmap showing hierarchical clustering of serum miRNA abundance data in all samples. The scale bar represents row Z‐scores with red and blue indicating higher and lower than mean abundance, respectively. Principal component analysis of serum miRNA abundance data with samples labelled according to either (*D*) experimental group, or (*E*) age.

Given that we observed close to normal levels of dystrophin in the heterozygote *mdx* mice, we were motivated to explore other genetic models with reduced dystrophin expression. The *Xist* ^Δhs^ mouse carries a mutation in the promoter of the *Xist* gene (encoding the long non‐coding RNA transcript responsible for the initiation of XCI,^45^) which leads to skewed XCI.^46^ Male *mdx* mice were crossed with female *Xist* ^Δhs^ mice^46^ to generate female F1 progeny (*mdx*‐*Xist* ^Δhs^) which carry a mutant DMD allele inherited from the male parent and a wild‐type DMD allele that is preferentially silenced by XCI inherited from the female parent. As such, these *mdx‐Xist* ^Δhs^ mice express varying levels of dystrophin in a random mosaic pattern across myonuclei.^33^ Serum was harvested repeatedly over an 11‐month period at 4, 8, 12, 16, 20, 24, 28, 32, 40, and 48 weeks of age by non‐terminal tail vein collections (*Figure* 2A). Female C57, non‐dystrophic *Xist* ^Δhs^, and dystrophic *mdx* mice were harvested as controls in parallel (all *n* = 5). At 78 weeks of age, mice were sacrificed, and dystrophin protein expression was determined in the quadriceps femoris muscles by Western blot (*Figure* 2B).^47^ Each *mdx‐Xist* ^Δhs^ mouse was then retrospectively assigned to low (8.4–12.9%, *n* = 5), medium (18.8–21%, *n* = 5), or high (32.4–43.5%, *n* = 5) dystrophin‐expressing groups (*Figure* 2A and 2B). While the *mdx‐Xist* ^Δhs^ mice exhibited high inter‐animal variability in dystrophin expression, dystrophin was previously shown to be expressed at similar levels between skeletal muscles within a single animal.^33^

### 3.3 Longitudinal analysis of serum miRNAs in mice expressing varying levels of dystrophin protein

To investigate the relationship between dystrophin and ex‐miRNA levels, we measured serum miRNA abundance utilizing the FirePlex methodology in all samples (i.e. low, medium, and high dystrophin expressing *mdx‐Xist* ^Δhs^ mice, and C57, *Xist* ^Δhs^ and *mdx* controls, ages: 4–48 weeks). This technique enables ex‐miRNA capture from crude serum using probe‐containing hydrogel particles that are subsequently quantified by flow cytometry. A custom panel of 17 miRNAs was designed based on a preliminary FirePlex profiling analysis (Supplementary Results and Discussion, *Figure* S1) and included previously identified miRNA biomarkers of high interest (miR‐1a‐3p, miR‐133a/b‐3p, miR‐206‐3p, miR‐193b‐3p, miR‐22‐3p, miR‐30a‐5p, and miR‐483‐3p,^26^, ^27^, ^28^, ^29^, ^30^, ^48^) putative cardiomiR biomarkers (miR‐208a‐3p, miR‐370‐3p, and miR‐539‐5p,^49^) and reference miRNAs for normalization and quality control (Supplementary File S1). miRNA analysis was performed on the FirePlex platform (*n* = 291), and signal intensities for the full FirePlex dataset are provided in Supplementary File S1. FirePlex profiling quality control analyses are described in Supplementary Results and Discussion and *Figure* S2.

Heatmap visualization of normalized ex‐miRNA abundance levels revealed a clear difference between the non‐dystrophic controls (C57 and *Xist* ^Δhs^) and the dystrophin‐deficient strains (*mdx* and *mdx‐Xist* ^Δhs^) (*Figure* 2C). Hierarchical clustering identified four groups of miRNAs: (i) the classical myomiRs (miR‐1a‐3p, miR‐133a/b‐3p, and miR‐206‐3p) and miR‐378a‐3p, which were significantly increased in the dystrophin‐deficient samples, (ii) miR‐483‐3p and the putative cardiomiRs (miR‐370‐3p, miR‐208a‐3p, and miR‐539‐5p), which exhibited higher inter‐group variability, (iii) other putative myomiR biomarkers (miR‐193b‐3p, miR‐22‐3p, and miR‐30a‐5p), which exhibited less clearly defined expression patterns but were generally elevated in dystrophin‐deficient serum samples, and (iv) quality control miRNAs (for data normalization and hemolysis assessment).

Principal component analysis showed that samples primarily clustered according to whether they were from wild‐type or dystrophin‐deficient groups (*Figure* 2D). Interestingly, the C57 and *Xist* ^Δhs^ controls clustered together, and samples from *mdx* and *mdx‐Xist* ^Δhs^ mice were mostly intermingled. There was no obvious age‐associated sample separation apparent from the PCA biplot (*Figure* 2E).

**Figure 3:**
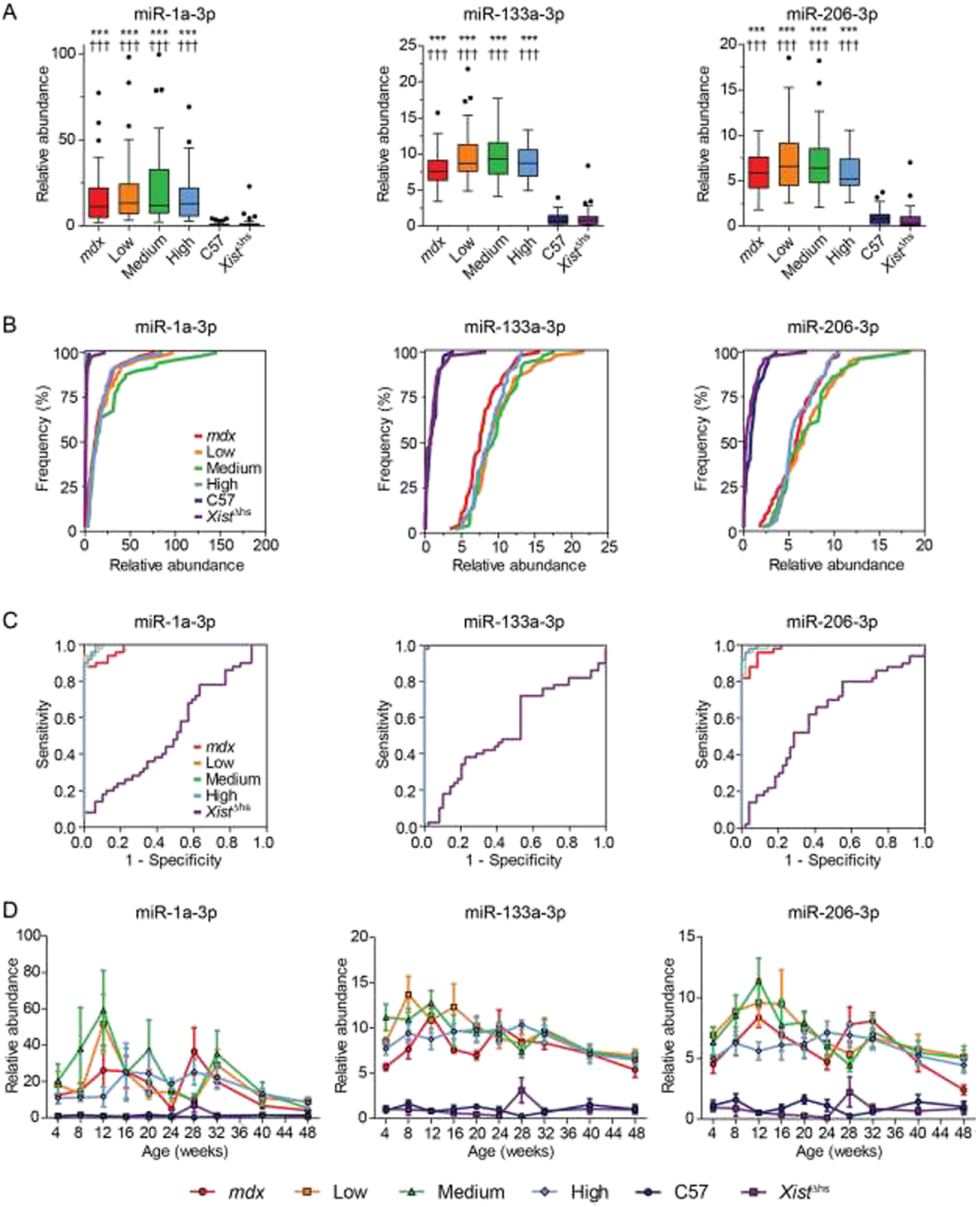
Serum myomiRs are elevated irrespective of dystrophin protein levels in *mdx‐Xist* ^Δhs^ mice. Serum miRNA abundance data for selected individual miRNAs (miR‐1a‐3p, miR‐133a‐3p, and miR‐206‐3p) measured using the FirePlex methodology. Data are shown as (*A*) Tukey box plots of all measurements pooled for each mouse strain (*n* ≥ 46 measurements, range: 46–50), (*B*) cumulative distribution function plots comparing experimental groups, (*C*) receiver operating characteristic curves to assess the potential of each miRNA to distinguish experimental groups from the C57 controls, and (*D*) time course plots of the mean for every group at each age over the full 4–48‐week time period. FirePlex miRNA data were normalized to the geometric average of miR‐16‐5p, miR‐17‐5p, and miR‐92a‐3p. Abundance ratios were scaled such that the mean of the C57 control was returned to a value of 1. Values in (*D*) are mean ± standard error of the mean. ***P < 0.001 for comparison with C57 group. †††*P* < 0.001 for comparison with the *Xist* ^Δhs^ group. *P*‐values are for Kruskal‐Wallis one‐way analysis of variance with Dunn's *post hoc* test.

**Figure 4:**
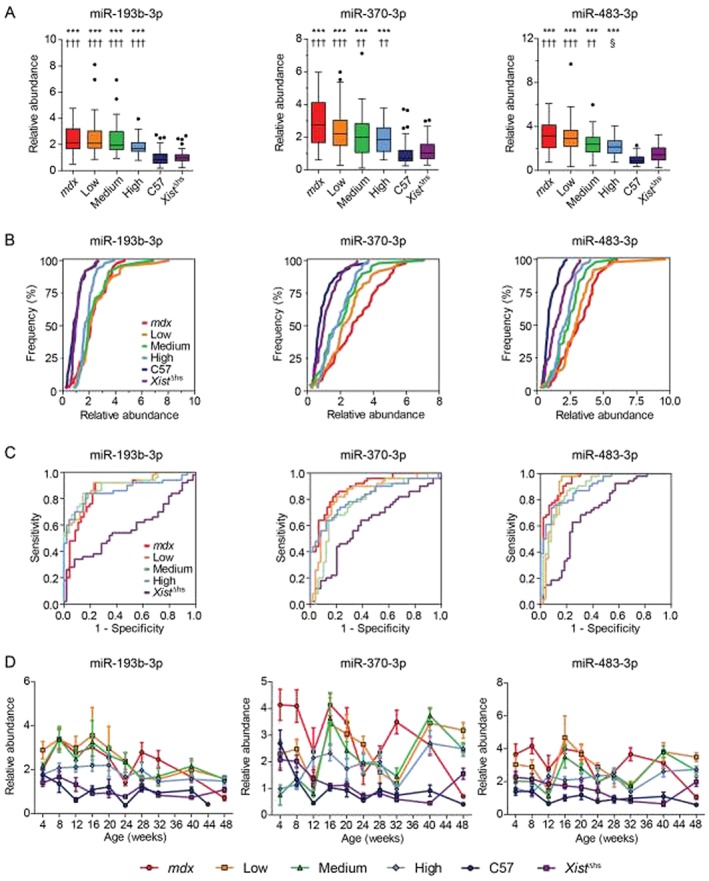
Dystrophin‐dependent reductions in serum levels of miR‐193b‐3p, miR‐370‐3p, and miR‐483‐3p in *mdx‐Xist* ^Δhs^ mice. Serum miRNA abundance data for selected individual miRNAs (miR‐193b‐3p, miR‐370‐3p, and miR‐483‐3p) measured using the FirePlex methodology. Data are shown as (*A*) Tukey box plots of all measurements pooled for each mouse strain (*n* ≥ 46 measurements, range: 46–50), (*B*) cumulative distribution function plots comparing experimental groups, (C) receiver operating characteristic curves to assess the potential of each miRNA to distinguish experimental groups from the C57 controls, and (D) time course plots of the mean for every group at each age over the full 4–48‐week time period. FirePlex miRNA data were normalized to the geometric average of miR‐16‐5p, miR‐17‐5p, and miR‐92a‐3p. Abundance ratios were scaled such that the mean of the C57 control was returned to a value of 1. Values in (D) are mean ± standard error of the mean. ***P < 0.001 for comparisons to the C57 group. ††*P* < 0.01, †††*P* < 0.001 for comparisons to the *Xist* ^Δhs^ group. §*P* < 0.05 for comparisons to the *mdx* group. *P*‐values are for Kruskal‐Wallis one‐way analysis of variance with Dunn's *post hoc* test.

Data for each individual miRNA were then considered separately (*Figures* 3 and 4), whereby abundance ratios were aggregated over all ages and visualized by (A) box plots, (B) CDF plots, (C) ROC plots to assess the potential of each putative miRNA biomarker to discriminate test groups from the C57 control, or (D) a time course plot of miRNA abundance ratios separated by both age and mouse strain.

The myomiRs (miR‐1a‐3p, miR‐133a‐3p, and miR‐206‐3p) (*Figure* 3) are the most commonly reported DMD miRNA biomarkers.^21^ FirePlex profiling showed a significant increase in serum abundance for these miRNAs in all dystrophin‐deficient strains that exhibited a tendency to decline towards wild‐type levels at the older ages (*Figure* 3A and 3D), consistent with our previous observations.^29^, ^31^ Interestingly, the box plots and CDF plots showed a clear difference between the wild‐type control strains and the dystrophin‐deficient strains, but there was no distinction between *mdx* mice and the three *mdx‐Xist* ^Δhs^ groups expressing different levels of dystrophin (*Figure* 3A and 3B). ROC curve analysis for the myomiRs showed near perfect discrimination between dystrophic and wild‐type animals (*Figure* 3C). Area under the curve values for all miRNA ROC curve analyses are provided in *Figure* S3. Notably, miR‐1a‐3p levels in dystrophin‐deficient animals exhibited the greatest variation between samples of these myomiRs (*Figure* 3A and 3D).

In contrast, serum abundance of miR‐193b‐3p, miR‐370‐3p, and miR‐483‐3p was found to be inversely related to the level of muscle dystrophin expression in the *mdx‐Xist* ^Δhs^ mice (*Figure* 4A and 4B). These three miRNAs were significantly elevated in the dystrophin‐deficient groups relative to wild‐type controls, although the magnitude of the difference observed was generally less than for the myomiRs (*Figure* 4A and 4D). For miR‐483‐3p, levels in the high dystrophin‐expressing group were significantly lowered (*P* < 0.05) relative to the *mdx* group. While this was the only example of a statistical difference between the mean abundance values for the intermediate dystrophin‐expressing groups, likely on account of the inherent high level of noise in these measurements, the association between ex‐miRNA levels and dystrophin expression level was clearly apparent form the CDF plot (*Figure* 4B). ROC curve analysis showed that these miRNAs exhibited reduced power to discriminate between healthy and affected mice (*Figure* 4C) relative to the myomiR group, described above (*Figure* 3C).

Data for the remaining miRNAs investigated are described in full in Supplementary Results and Discussion, *Figures* S4 and S5 and are not further discussed here (these miRNAs either behave in similar ways to those already discussed or are not otherwise interesting). FirePlex data were validated using an orthogonal methodology, RT‐qPCR, at two time points (16 and 24). Results were very similar between the two techniques, confirming our biological findings and demonstrating the technical robustness of our profiling strategy (Supplementary Results and Discussion, *Figures* S6 and S7). Similarly, as the animals used in the FirePlex screen were necessarily female mice, we sought to further validate findings for key miRNAs in male mice. To this end, miRNA expression was measured by RT‐qPCR in a new set of C57 and *mdx* mice (both male mice and female mice) at four ages (10, 24, 52, and 80 weeks). The results were largely similar between sexes, whereby the putative miRNA biomarkers were consistently elevated in the *mdx* mice. However, the male *mdx* mice exhibited a peak in ex‐miRNA levels at 24 weeks of age that was absent in female *mdx* serum, and the magnitude of ex‐miRNA elevation was generally higher in male *mdx* serum (Supplementary Results and Discussion and *Figure* S8). A decline in overall ex‐miRNA abundance levels at the later ages in *mdx* mice was observed for all miRNAs tested. (Minor validation discrepancies and negative data are further described in Supplementary Results and Discussion, and *Figures* S9 and S10).

### 3.4 Uniform dystrophin expression is associated with serum miRNA restoration

The observation that serum myomiR levels were not influenced by differences in muscle dystrophin expression in *mdx‐Xist* ^Δhs^ mice was surprising, given that (i) previous studies have reported restoration of serum myomiR abundance to wild‐type levels following partial dystrophin restoration in animal models^26^, ^28^, ^29^, ^30^, ^32^ (ii) we previously showed that the level of serum myomiR restoration is commensurate with the level of exon skipping‐mediated dystrophin rescue^30^ (iii) the levels of dystrophin expressed in the muscles of *mdx‐Xist* ^Δhs^ mice are similar to that of exon skipping‐treated *mdx* muscles; and (iv) BMD patients have been reported to exhibit intermediate serum myomiR levels between those of DMD patients and healthy controls.^26^, ^50^, ^51^ One possible explanation for this apparent paradox is the uniform vs. non‐uniform patterns of dystrophin protein distribution in the exon skipping‐treated *mdx* group relative to *mdx‐Xist* ^Δhs^ mice.^33^ To test this possibility, we sought to directly compare exon skipping‐treated *mdx* mice with *mdx‐Xist* ^Δhs^ at the same age. Male *mdx* mice were injected with a single 12.5 mg/kg dose of a PPMO conjugate via the tail vein at age 6 weeks and sacrificed 2 weeks later.

**Figure 5:**
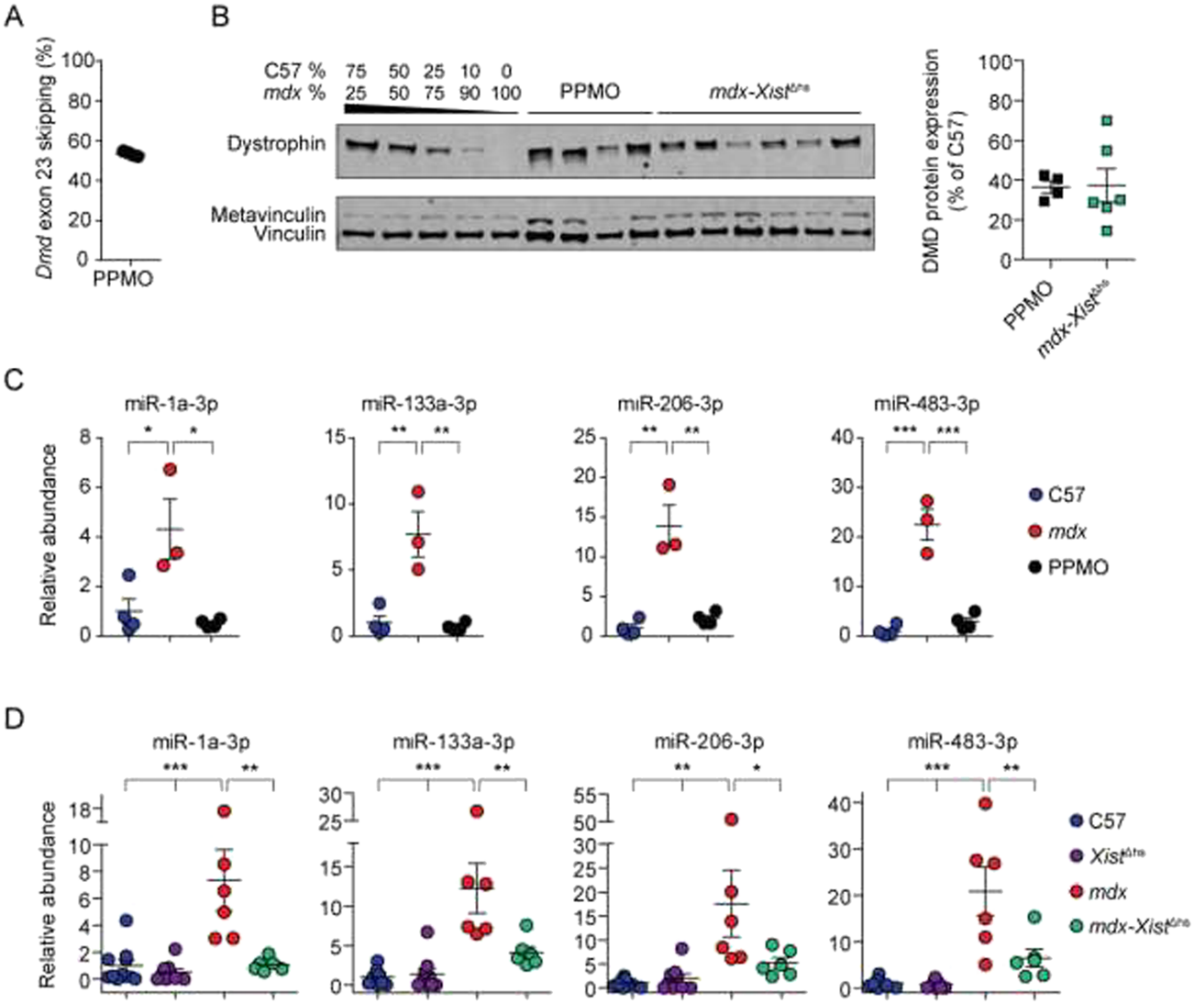
ex‐miRNA levels in PPMO‐treated *mdx* and *mdx‐Xist* ^Δhs^ serum. (*A*) Dystrophin exon 23 skipping in the tibialis anterior of peptide‐phosphorodiamidate morpholino oligomer (PPMO)‐treated *mdx* (*n* = 4) mice was confirmed by RT‐qPCR. (*B*) Western blot analysis of dystrophin protein expression in tibialis anterior muscles from C57, *mdx*, PPMO‐treated *mdx*, and *mdx‐Xist* ^Δhs^ mice. Vinculin was used as a loading control. A standard curve, consisting of mixtures of C57 and *mdx* protein lysates in ratios as indicated, was used to quantify dystrophin protein expression in the PPMO‐treated *mdx* and *mdx‐Xist* ^Δhs^ samples. ex‐miRNA abundance was measured by small RNA TaqMan RT‐qPCR in (*C*) serum from male C57 (*n* = 4), *mdx* (*n* = 3), and PPMO‐treated *mdx* (*n* = 4) mice, and (*D*) female C57 (*n* = 10), *Xist* ^Δhs^ (*n* = 9), *mdx* (*n* = 6), and *mdx‐Xist* ^Δhs^ (*n* = 6). All mice were 8‐week old. qPCR data were normalized to a synthetic spike‐in control oligonucleotide (cel‐miR‐39). Abundance ratios were scaled such that the mean of the C57 control was returned to a value of 1. All values are mean ± standard error of the mean. *P < 0.05, **P < 0.01, ***P < 0.001, for one‐way analysis of variance with Tukey *post hoc* test.

Mean exon skipping efficiency was determined to be 53.5% by RT‐qPCR (*Figure* 5A), resulting in restoration of dystrophin protein to an average of 36.5% of wild‐type levels as assessed by Western blot (*Figure* 5B). In parallel, dystrophin expression was quantified in 8‐week‐old *mdx‐Xist* ^Δhs^ mice (*n =* 6) from a new litter. These mice were selected based on the high levels of dystrophin protein expression (average of 37.4% of wild‐type, Figure 5B) that were similar to those achieved after exon skipping and comparable to the high dystrophin expressing group in the first analysis described above (*Figure* 2A). ex‐miRNA levels were determined in the serum of PPMO‐treated animals (*n* = 4) and compared with untreated *mdx* (*n* = 3) and C57 wild‐type controls (*n* = 4). Circulating miRNA levels were completely restored to wild‐type levels in all treated animals (*Figure* 5C) consistent with previous reports.^28^, ^29^, ^30^ In contrast, ex‐miRNA levels in *mdx‐Xist* ^Δhs^ (*n* = 6) mice were present at intermediate abundance levels between those of wild‐type [i.e. C57 (*n* = 10) and *Xist* ^Δhs^ (*n* = 9)] and *mdx* (*n* = 6) controls in the cases of miR‐133a‐3p, miR‐206‐3p, and miR‐483‐3p (*Figure* 5D).

**Figure 6:**
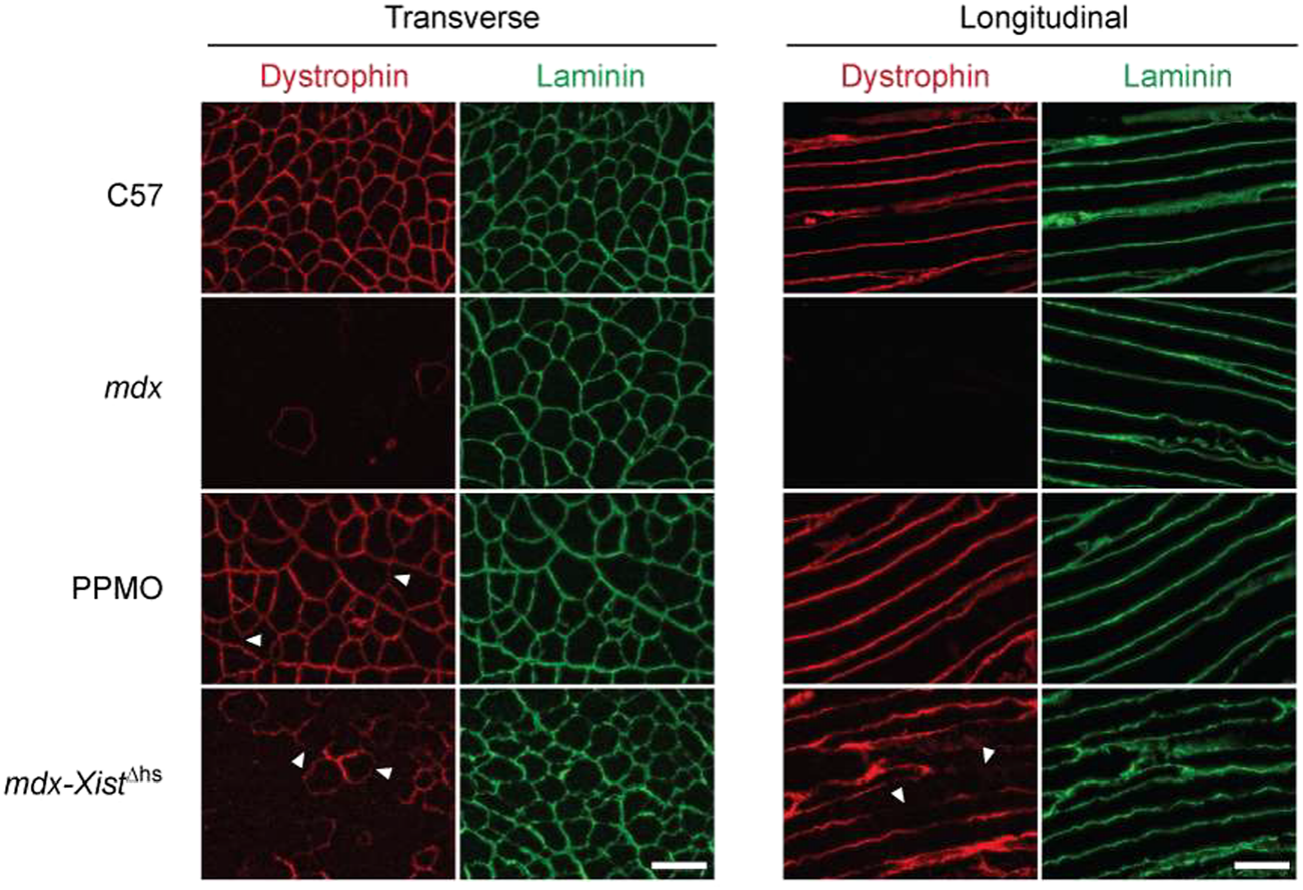
Dystrophin protein localization in peptide‐phosphorodiamidate morpholino oligomer (PPMO)‐treated *mdx* and *mdx‐Xist* ^Δhs^ muscle. Representative immunofluorescence staining of dystrophin protein in transverse and longitudinal tibialis anterior sections for C57, *mdx*, PPMO‐treated *mdx*, and *mdx‐Xist* ^Δhs^ mice (expressing ~40% of wild‐type dystrophin protein). Sections were co‐stained with laminin to label myofibers. Arrowheads indicate regions of sarcolemma with weaker dystrophin staining. Scale bars represent 100 μm, images taken at ×20 magnification.

Given that the restoration of ex‐myomiR abundance that was observed following PPMO treatment was not recapitulated in the high *mdx‐Xist* ^Δhs^ group (*Figure* 3), despite these animals expressing similar levels of total dystrophin protein, we next sought to compare the pattern of sarcolemmal dystrophin expression in these two models. Dystrophin protein localization was visualized in TA muscle of PPMO‐treated animals and *mdx‐Xist* ^Δhs^ mice at 8 weeks in both transverse and longitudinal orientations. We observed a uniform pattern of dystrophin in the PPMO‐treated animals (*Figure* 6), although there were small regions of less intense dystrophin staining compared to controls in some places (see arrowheads in *Figure* 6). Conversely, TA muscles from the *mdx‐Xist* ^Δhs^ mice exhibited a patchy, non‐homogeneous distribution of dystrophin in both transverse and longitudinal sections, whereby incomplete staining at the periphery of each fiber was observed for (*Figure* 6). Inspection of transverse sections appeared to show some degree of between‐fiber patchiness, but all fibers appeared to be dystrophin positive when viewed in longitudinal orientation, instead indicative of within‐fiber patchiness. Similar results were obtained for the dystrophin‐associated protein complex components dystrobrevin alpha and neuronal nitric oxide synthase 1, whereby uniform expression was restored after PPMO treatment, and patchy staining was observed in the *mdx‐Xist* ^Δhs^ model (*Figure* S11).

**Figure 7:**
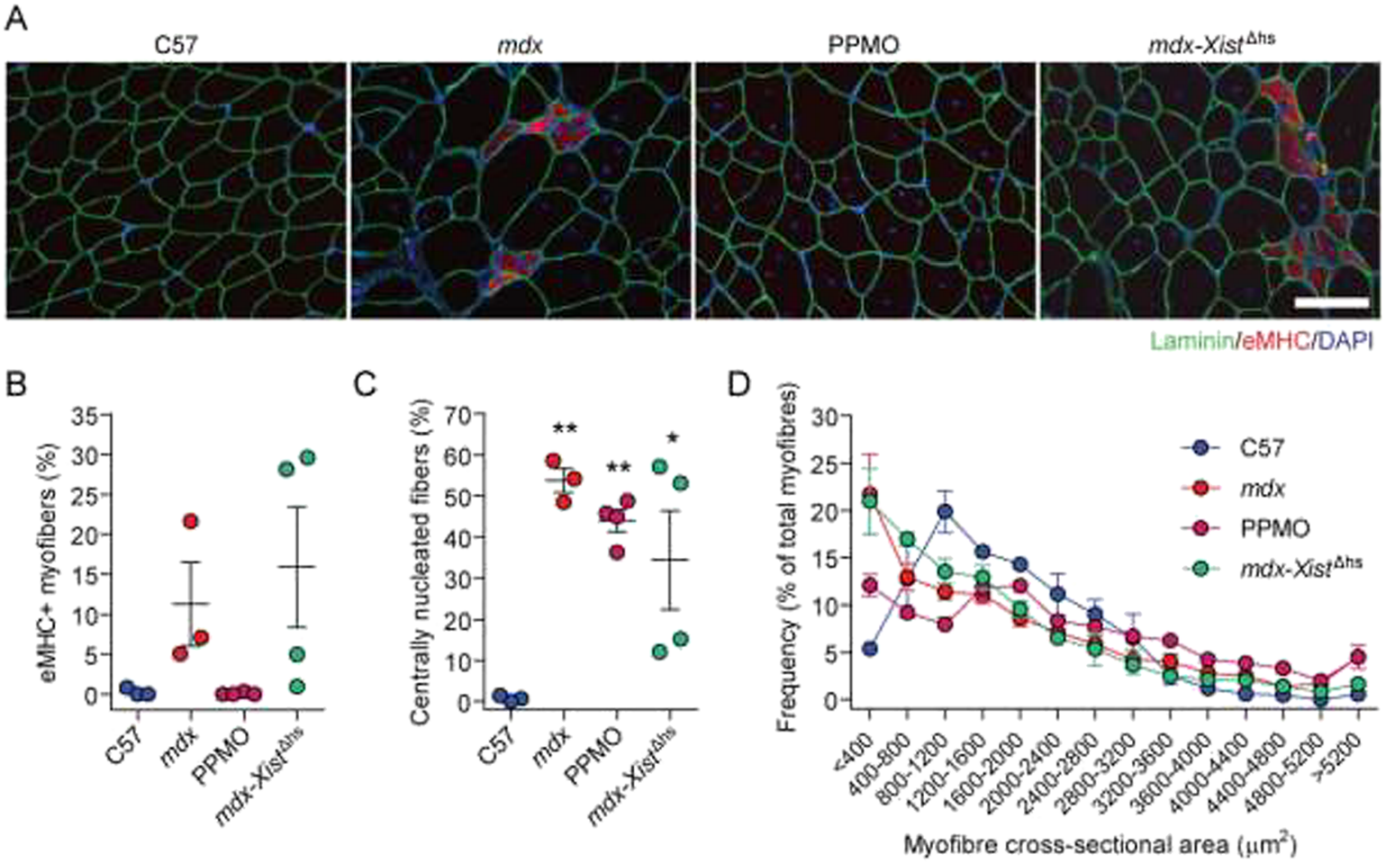
Uniform dystrophin protein expression is required to stabilize myofiber turnover in dystrophic muscle. (*A*) Representative immunofluorescence staining for embryonic myosin heavy chain (eMHC) in transverse tibialis anterior muscle sections for C57, *mdx*, peptide‐phosphorodiamidate morpholino oligomer (PPMO)‐treated *mdx*, and *mdx‐Xist* ^Δhs^ mice. Sections were co‐stained with laminin to label myofibers and DAPI to stain nuclei. Muscle regeneration was quantified by (*B*) percentage of eMHC positive myofibers, (*C*) percentage of centrally nucleated fibers, and (*D*) myofiber cross‐sectional area. All values are mean ± standard error of the mean. *P < 0.05, **P < 0.01 for one‐way analysis of variance with Tukey *post hoc* test. Scale bars represent 100 μm, images taken at ×20 magnification.

Assessment of muscle regeneration in these mice revealed that eMHC positive myofibers were present in the *mdx* and *mdx‐Xist* ^Δhs^ animals, indicative of active regeneration occurring sporadically at a small number of foci (*Figure*s 7A and 7B). In contrast, PPMO‐treated *mdx* muscles were almost completely devoid of eMHC positive fibers (*Figures* 7A and 7B). Levels of historic muscle regeneration, as determined by the percentage of centrally nucleated fibers, were similar between the *mdx*, PPMO‐treated *mdx*, and *mdx‐Xist* ^Δhs^ groups (*Figure* 7C). Similarly, the numbers of smaller fibers (i.e. immature and regenerating myofibers) were increased in the *mdx* and *mdx‐Xist* ^Δhs^ animals (*Figure* 7D). In contrast, the number of smaller fibers was reduced in the PPMO‐treated *mdx* group relative to untreated *mdx* controls, and fiber hypertrophy was observed in all dystrophin‐deficient groups (*Figure* 7D). These results suggest that active muscle regeneration is occurring in both the *mdx* and *mdx‐Xist* ^Δhs^ groups but not in the PPMO‐treated *mdx* group, whereby myofiber turnover is stabilized as a consequence of exon skipping‐mediated dystrophin restoration. Additionally, expression from birth of low levels of unevenly distributed dystrophin protein is insufficient to prevent myofiber turnover, as exemplified by the increase in centrally nucleated fibers relative to C57 controls. Taken together, these findings highlight that the pattern of dystrophin distribution at the sarcolemma is an important factor that contributes to myofiber stability and thereby also to the release of ex‐miRNAs into the circulation.

## 4 Discussion

In this study, we sought to investigate the relationship between skeletal muscle dystrophin protein levels and the serum abundance of ex‐miRNAs, with respect to their potential as pharmacodynamic biomarkers. Initially, we investigated *mdx* heterozygotes, although near‐to‐normal levels of dystrophin protein limited the usefulness of this model. We therefore utilized a DMD mouse model (*mdx‐Xist* ^Δhs^) expressing varying levels of dystrophin protein from birth that are similar to the levels achieved by exon skipping in the *mdx* mouse.

We have previously shown that increased dystrophin protein levels led to improved muscle function, life span, and CK levels in these mice.^33^, ^52^, ^53^ Upon measurement of ex‐miRNA abundance in serum, we unexpectedly observed that the myomiRs were consistently elevated in all *mdx‐Xist* ^Δhs^ groups, irrespective of the level of dystrophin expressed in skeletal muscle. This is in direct contrast with what has been observed in PPMO‐treated mice, whereby similar levels of dystrophin restoration by exon skipping treatment were associated with complete restoration of ex‐myomiR abundance to wild‐type levels.^29^, ^30^ We further investigated this phenomenon by comparing the distribution of dystrophin protein expression between *mdx‐Xist* ^Δhs^ and PPMO*‐*treated *mdx* mice and observed that dystrophin protein was uniformly distributed at the sarcolemma in PPMO‐treated animals, whereas *mdx‐Xist* ^Δhs^ mice exhibited a patchy, non‐homogeneous pattern, whereby dystrophin staining at the sarcolemma was discontinuous within a myofiber.

While PPMO‐mediated dystrophin restoration resulted in a cessation of myofiber turnover and a reduction in circulating miRNA biomarkers to wild‐type levels, it will be interesting to determine if this is also the case for other types of therapeutic intervention. For example, up‐regulation of the dystrophin paralog utrophin would be expected to functionally compensate for the absence of dystrophin and thereby stabilize myofiber turnover in a similar manner to that observed after exon skipping. As such, ex‐miRNAs might also have utility as pharmacodynamic biomarkers for utrophin‐activating therapies. In support of this notion, the *mdx*‐Fiona mouse (which overexpresses utrophin on a dystrophin‐deficient background^54^) exhibits wild‐type serum abundance levels for the key myomiR biomarkers (Kay Davies, personal communication). Utrophin is known to already be up‐regulated in *mdx* muscle,^55^ which may contribute to the relatively mild pathology observed in this model but also implies that this level of utrophin expression is not enough to prevent ex‐miRNA release.

The patchy sarcolemmal staining observed in *mdx‐Xist* ^Δhs^ mice is consistent with the myonuclear domain hypothesis,^56^ whereby each myonucleus serves its proximal myofiber cytoplasmic territory. Accordingly, these data suggest that diffusion of dystrophin mRNA and protein is subject to some degree of spatial restriction, as has been suggested more generally in other contexts.^57^ The resulting segmented pattern of dystrophin expression is unlikely to be fully sufficient to protect a myofiber from degeneration, as contractile damage (leading to fiber death) will still occur at non‐protected, dystrophin‐negative regions of the sarcolemma. Similar effects have been reported in terms of between‐myofiber mosaicism. Low levels of dystrophin expressed in the majority of myofibers were found to result in an improvement in dystrophic pathology, whereas mice expressing high levels of dystrophin in a smaller number of fibers still exhibited typical dystrophic features.^58^, ^59^

The goal of this study was to investigate the relationship between dystrophin protein expression and ex‐miRNA biomarker levels using genetic models. However, the non‐uniform pattern of dystrophin expression observed in the *mdx‐Xist* ^Δhs^ mice is different from the uniform expression pattern that is observed following exon skipping or gene therapy, which constitutes a limitation of this model in these contexts. In contrast, the findings presented here have potential implications for other dystrophin re‐expression strategies such as CRISPR/Cas9‐mediated gene editing and cell‐based therapeutics.^60^, ^61^ While the former approach has yielded impressive results *in vivo*,^62^, ^63^ it will most likely be less efficacious in humans due to the requirement for enormous viral titers, possible immune responses to Cas9 protein^64^ or pre‐existing immunity to adeno‐associated virus,^65^ and difficulties associated with repeated administration.^66^ Importantly, suboptimal CRISPR treatment would likely lead to fiber mosaicism, with parts of the myofiber positive and other parts negative for dystrophin, due to incomplete editing of all myonuclei. Similarly, current strategies for cell‐based therapies involve transplantation of dystrophin‐expressing myoblasts that subsequently fuse with existing dystrophin‐negative myofibers in the host muscle, thereby also generating chimeric fibers.^67^ The expected outcomes of these two therapeutic strategies are therefore approximated by the XCI‐driven non‐homogeneous dystrophin expression pattern observed in the *mdx‐Xist* ^Δhs^ mouse. As such, our findings highlight an important potential limitation of these therapeutic approaches; as incomplete, non‐homogeneous dystrophin re‐expression may be insufficient to protect myofibers from contractile damage. Consistent with this hypothesis, a subset of BMD patients exhibits non‐homogeneous staining at the sarcolemma, which is correlated with a more severe disease manifestation.^68^

Despite observing that myomiR levels were consistently up‐regulated in *mdx‐Xist* ^Δhs^ serum, we also identified several miRNAs that exhibited dystrophin‐dependent reductions in serum abundance levels. For example, miR‐483‐3p shows promise as a pharmacodynamic biomarker of exon skipping efficacy (which we recently identified in a small RNA‐sequencing study in *mdx* serum^29^) and was found to be inversely related to dystrophin protein levels in *mdx‐Xist* ^Δhs^ mice. The observation that distinct relationships exist between dystrophin expression in muscle and the serum levels of the various miRNAs suggests that multiple mechanisms contribute to ex‐miRNAs into the circulation.

In conclusion, these data suggest that ex‐myomiR abundance is a function of both total dystrophin levels and the pattern of dystrophin distribution at the sarcolemma. These findings have implications for therapeutic strategies that result in the generation of heterokaryotic myofibers, such as CRISPR‐mediated dystrophin correction and cell therapy.

## Conflict of interest

MJAW is a founder of Pepgen Ltd, which aims to commercialize peptide technology similar to that utilized in this manuscript. AA‐R. discloses being employed by LUMC which has patents on exon skipping technology, some of which has been licensed to BioMarin and subsequently sublicensed to Sarepta. As co‐inventor of some of these patents, AA‐R. is entitled to a share of royalties. AA‐R. further discloses being *ad hoc* consultant for PTC Therapeutics, Summit PLC, Alpha Anomeric, BioMarin Pharmaceuticals Inc., Eisai, Global Guidepoint and GLG consultancy, Grunenthal, Wave and BioClinica having been a member of the Duchenne Network Steering Committee (BioMarin) and being a member of the scientific advisory boards of ProQR and Philae Pharmaceuticals. Remuneration for these activities is paid to LUMC. LUMC also received speaker honoraria from PTC Therapeutics and BioMarin Pharmaceuticals and funding for contract research from Italpharmaco and Alpha Anomeric. The remaining authors declare no competing interests.

## Author's contributions

This work was conceived by TCR, AA‐R, MvP and MJAW. Experiments were performed by TLEvW, YL, AMLC‐S, CA, AB, MH, LEC, GM, MvP, and TCR. The first draft of the manuscript was written by TLEvW and TCR. All authors contributed to, and approved, the final manuscript.

## Supporting information

Table S1. Small RNA TaqMan assays used for miRNA quantification.
Table S2. Primer and probe sequences used for quantifying exon skipping.
Figure S1. Profiling of differentially abundant ex‐miRNAs in dystrophic serum.
Figure S2. Serum miRNA profiling quality control analyses.
Figure S3. Area under the curve values for ROC curve analysis.
Figure S4. Analysis of individual serum miRNAs: miR‐208a‐3p, miR‐378a‐3p and miR‐539‐5p.
Figure S5. Analysis of individual serum miRNAs: miR‐133b‐3p, miR‐22‐3p and miR‐30a‐5p.
Figure S6. RT‐qPCR validation of serum miRNA abundance at 16 weeks of age.
Figure S7. RT‐qPCR validation of serum miRNA abundance at 24 weeks of age.
Figure S8. Differential ex‐miRNA abundance in dystrophic serum from male and female mice.
Figure S9 Failure to validate findings for lowly abundant serum miRNAs.
Figure S10. Profiling of differentially abundant ex‐miRNAs in aged dystrophic serum.
Figure S11. Localization of DTNA and NOS1 expression in PPMO‐treated *mdx* and *mdx‐Xist*Δhs mice.

Supporting info item

Supporting info item
