# Supplementary material for: Uniform sarcolemmal dystrophin expression is required to prevent extracellular microRNA release and improve dystrophic pathology
Source: J Cachexia Sarcopenia Muscle. 2019 Dec 17;11(2):578–93. doi: 10.1002/jcsm.12506 (PMC7113513; doi:10.1002/jcsm.12506)
Supplement: Supplementary file 1 — Table S1. Small RNA TaqMan assays used for miRNA quantification. Table S2. Primer and probe sequences used for quantifying exon skipping. Figure S1. Profiling of differentially abundant ex‐miRNAs in dystrophic serum. Figure S2. Serum miRNA profiling quality control analyses. Figure S3. Area under the curve values for ROC curve analysis. Figure S4. Analysis of individual serum miRNAs: miR‐208a‐3p, miR‐378a‐3p and miR‐539‐5p. Figure S5. Analysis of individual serum miRNAs: miR‐133b‐3p, miR‐22‐3p and miR‐30a‐5p. Figure S6. RT‐qPCR validation of serum miRNA abundance at 16 weeks of age. Figure S7. RT‐qPCR validation of serum miRNA abundance at 24 weeks of age. Figure S8. Differential ex‐miRNA abundance in dystrophic serum from male and female mice. Figure S9 Failure to validate findings for lowly abundant serum miRNAs. Figure S10. Profiling of differentially abundant ex‐miRNAs in aged dystrophic serum. Figure S11. Localization of DTNA and NOS1 expression in PPMO‐treated mdx and mdx‐XistΔhs mice. [file JCSM-11-578-s001.pdf]

Title

Uniform sarcolemmal dystrophin expression is required to prevent extracellular microRNA release and improve dystrophic pathology

Authors

Tirsa L. E. van Westering, Yulia Lomonosova, Anna M. L. Coenen-Stass, Corinne A. Betts, Amarjit Bhomra, Margriet Hulsker, Lucy E. Clark, Graham McClorey, Annemieke Aartsma-Rus, Maaike van Putten, Matthew J. A. Wood, and Thomas C. Roberts

miRNA	Assay ID	Target Sequence
**miR-1a-3p**	002222	UGGAAUGUAAAGAAGUAUGUAU
**miR-133a-3p**	002246	UUUGGUCCCCUUCAACCAGCUG
**miR-193b-3p**	002467	AACUGGCCCACAAAGUCCCGCU
**miR-206-3p**	000510	UGGAAUGUAAGGAAGUGUGUGG
**miR-208a-3p**	000511	AUAAGACGAGCAAAAAGCUUGU
**miR-370-3p**	002275	GCCUGCUGGGGUGGAACCUGGU
**miR-378-3p**	002243	ACUGGACUUGGAGUCAGAAGG
**miR-483-3p**	002560	UCACUCCUCCCCUCCCGUCUU
**miR-486a-5p**	001278	UCCUGUACUGAGCUGCCCCGAG
**miR-539-5p**	001286	GGAGAAAUUAUCCUUGGUGUGU
**cel-miR-39**	000200	UGUCAGUUUGUCAAAUACCCCA

Table S1

Small RNA TaqMan assays used for miRNA quantification.

All sequences are 5′ to 3′.

Target Gene		Sequence
Dmd exon 20-21	Forward	AGATGACAACTACTGCCGAA
	Reverse	GAAGAGCTGACAATCTGTTGAC
	Probe	AGTCTACCACCCTATCAGAGCCAACA
Dmd exon 23-24	Forward	GAAACTTTCCTCCCAGTTGGT
	Reverse	CAGGCCATTCCTCTTTCAGG
	Probe	TCAACTTCAGCCATCCATTTCTGTAAGGT

Table S2

Primer and probe sequences used for quantifying exon skipping.

All sequences are 5′ to 3′.

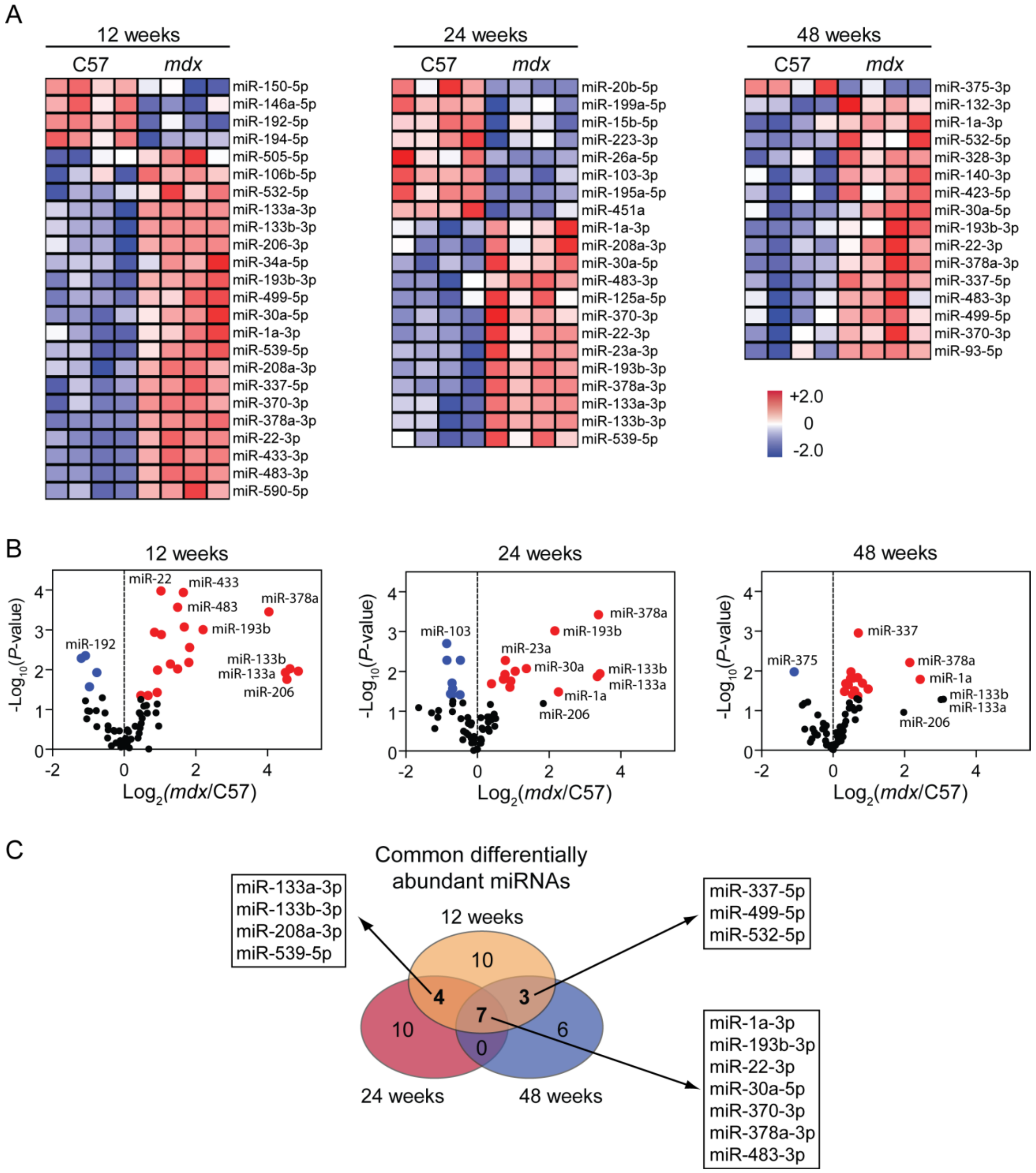

A
12 weeks
C57
mdx
miR-150-5p
miR-146a-5p
miR-192-5p
miR-194-5p
miR-505-5p
miR-106b-5p
miR-532-5p
miR-133a-3p
miR-133b-3p
miR-206-3p
miR-34a-5p
miR-193b-3p
miR-499-5p
miR-30a-5p
miR-1a-3p
miR-539-5p
miR-208a-3p
miR-337-5p
miR-370-3p
miR-378a-3p
miR-22-3p
miR-433-3p
miR-483-3p
miR-590-5p
24 weeks
C57
mdx
miR-20b-5p
miR-199a-5p
miR-15b-5p
miR-223-3p
miR-26a-5p
miR-103-3p
miR-195a-5p
miR-451a
miR-1a-3p
miR-208a-3p
miR-30a-5p
miR-483-3p
miR-125a-5p
miR-370-3p
miR-22-3p
miR-23a-3p
miR-193b-3p
miR-378a-3p
miR-133a-3p
miR-133b-3p
miR-539-5p
48 weeks
C57
mdx
miR-375-3p
miR-132-3p
miR-1a-3p
miR-532-5p
miR-328-3p
miR-140-3p
miR-423-5p
miR-30a-5p
miR-193b-3p
miR-22-3p
miR-378a-3p
miR-337-5p
miR-483-3p
miR-499-5p
miR-370-3p
miR-93-5p
+2.0
0
-2.0
B
12 weeks
-Log10(P-value)
Log2(mdx/C57)
miR-22
miR-433
miR-483
miR-378a
miR-193b
miR-192
miR-133b
miR-133a
miR-206
24 weeks
miR-378a
miR-193b
miR-103
miR-23a
miR-30a
miR-133b
miR-133a
miR-1a
miR-206
48 weeks
miR-337
miR-375
miR-378a
miR-1a
miR-133b
miR-133a
miR-206
C
Common differentially abundant miRNAs
12 weeks
24 weeks
48 weeks
10
10
6
4
3
7
0
miR-133a-3p
miR-133b-3p
miR-208a-3p
miR-539-5p
miR-337-5p
miR-499-5p
miR-532-5p
miR-1a-3p
miR-193b-3p
miR-22-3p
miR-30a-5p
miR-370-3p
miR-378a-3p
miR-483-3p

Figure S1

Profiling of differentially abundant ex-miRNAs in dystrophic serum.

The serum of C57 and *mdx* mice was analyzed using a custom FirePlex miRNA panel (68 miRNAs) at three ages (12, 24 and 48 weeks). FirePlex miRNA data were normalized to the geometric average of miR-16-5p, miR-17-5p and miR-92a-3p. Differentially abundant miRNAs ($P<0.05$, Student's *t*-test) were visualized by (A) heatmap and (B) volcano plot. The scale bar for the heatmap represents row Z-scores with red and blue indicating higher and lower than mean abundance respectively. Statistically significant changes are highlighted in red and blue (for elevated and lowered miRNAs respectively) on the volcano plots. (C) Venn diagram illustrating differentially abundant ex-miRNAs that are common between the different ages.

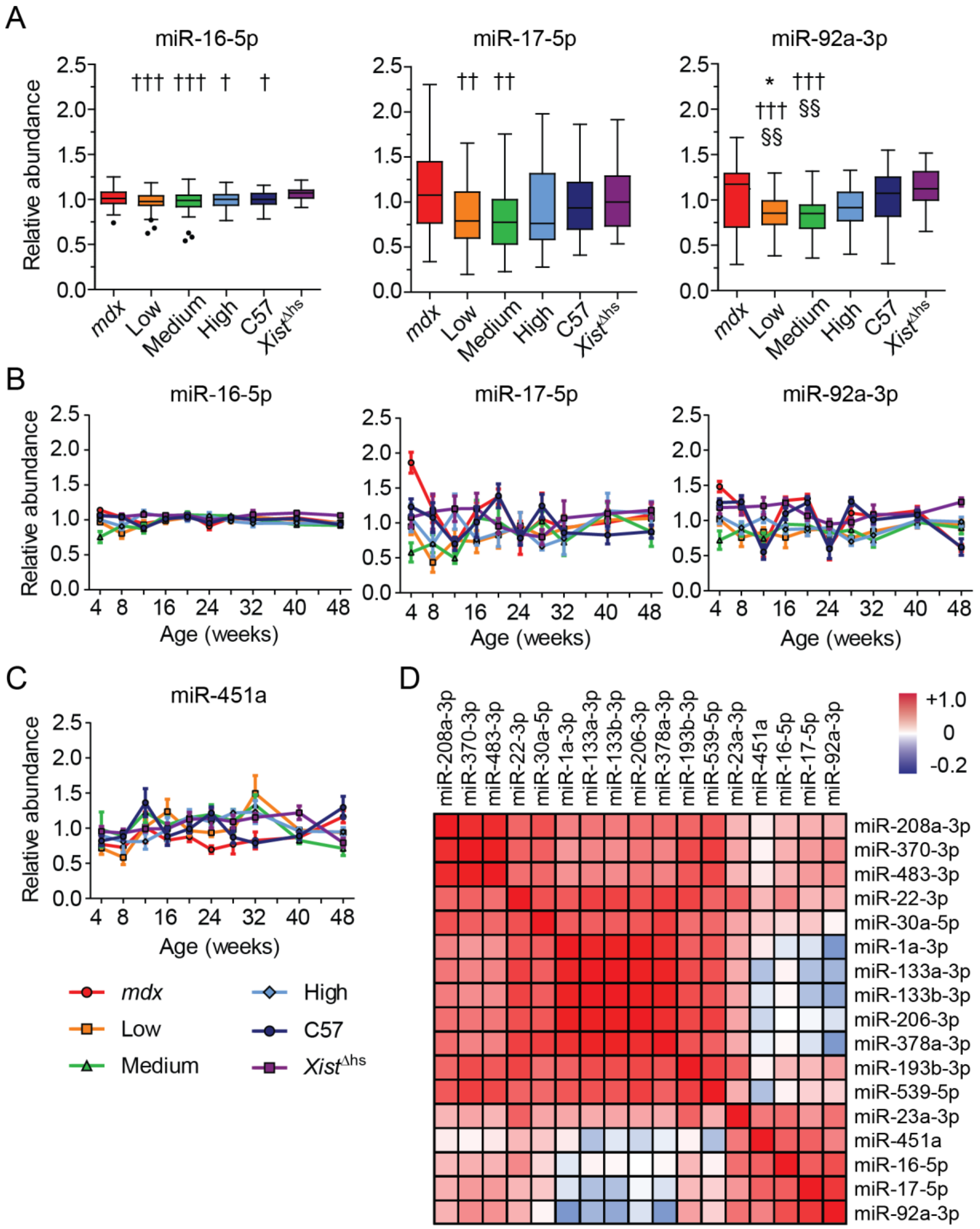

A
miR-16-5p
miR-17-5p
miR-92a-3p
Relative abundance
mdx
Low
Medium
High
C57
XistΔhs
B
Age (weeks)
C
miR-451a
D
+1.0
0
-0.2
miR-208a-3p
miR-370-3p
miR-483-3p
miR-22-3p
miR-30a-5p
miR-1a-3p
miR-133a-3p
miR-133b-3p
miR-206-3p
miR-378a-3p
miR-193b-3p
miR-539-5p
miR-23a-3p
miR-451a
miR-16-5p
miR-17-5p
miR-92a-3p

Figure S2

Serum miRNA profiling quality control analyses.

The miRNAs; miR-16-5p, miR-17-5p and miR-92a-3p, were selected as normalizer controls for the FirePlex data based on their stable abundance between samples (determined using the geNorm method). Serum levels (background subtracted but pre-normalization) of these normalizer miRNAs are shown in (A) Tukey box plots of all measurements pooled for each mouse strain ($n \geq 46$ measurements, range: 46-50), and (B) time course plots of the mean for every group at each age over the full 4-48 week period. *$P<0.05$ for comparisons to the C57 group. †$P<0.05$, ††$P<0.01$, †††$P<0.001$ for comparisons to the *Xist*$^{\Delta hs}$ group. §§$P<0.01$ for comparison to the *mdx* group. *P*-values are for one-way ANOVA with Tukey *post hoc* test. (C) Normalized serum abundance of miR-451a, an erythrocyte-enriched miRNA used as a marker of hemolysis, over the full time course. Values in (B) and (C) are mean ± SEM, $n=5$ in most cases. (D) Heatmap of Spearman's *r* correlation coefficients for unnormalized ex-miRNA abundance patterns measured in the full FirePlex screen.

	AUC				
	mdx	Low	Medium	High	*Xist*$^{\Delta hs}$
miR-1a-3p	0.97	0.99	0.98	0.98	0.55
miR-133a-3p	1.00	1.00	1.00	1.00	0.51
miR-133b-3p	1.00	1.00	1.00	1.00	0.50
miR-206a-3p	0.97	0.99	0.98	0.98	0.60
miR-193b-3p	0.82	0.81	0.80	0.77	0.59
miR-22-3p	0.84	0.85	0.82	0.85	0.61
miR-30a-5p	0.90	0.89	0.85	0.89	0.68
miR-378a-3p	1.00	1.00	1.00	1.00	0.57
miR-483-3p	0.89	0.86	0.80	0.82	0.71
miR-208a-3p	0.91	0.90	0.82	0.88	0.77
miR-370-3p	0.84	0.79	0.71	0.75	0.63
miR-539-5p	0.89	0.83	0.82	0.83	0.58

Figure S3

Area under the curve values for ROC curve analysis.

Area under the curve (AUC) values from ROC curve analysis comparing the ability of each ex-miRNA to discriminate between the experimental group and the C57 control. The table is color-coded, with red and blue indicating higher and lower AUC values respectively.

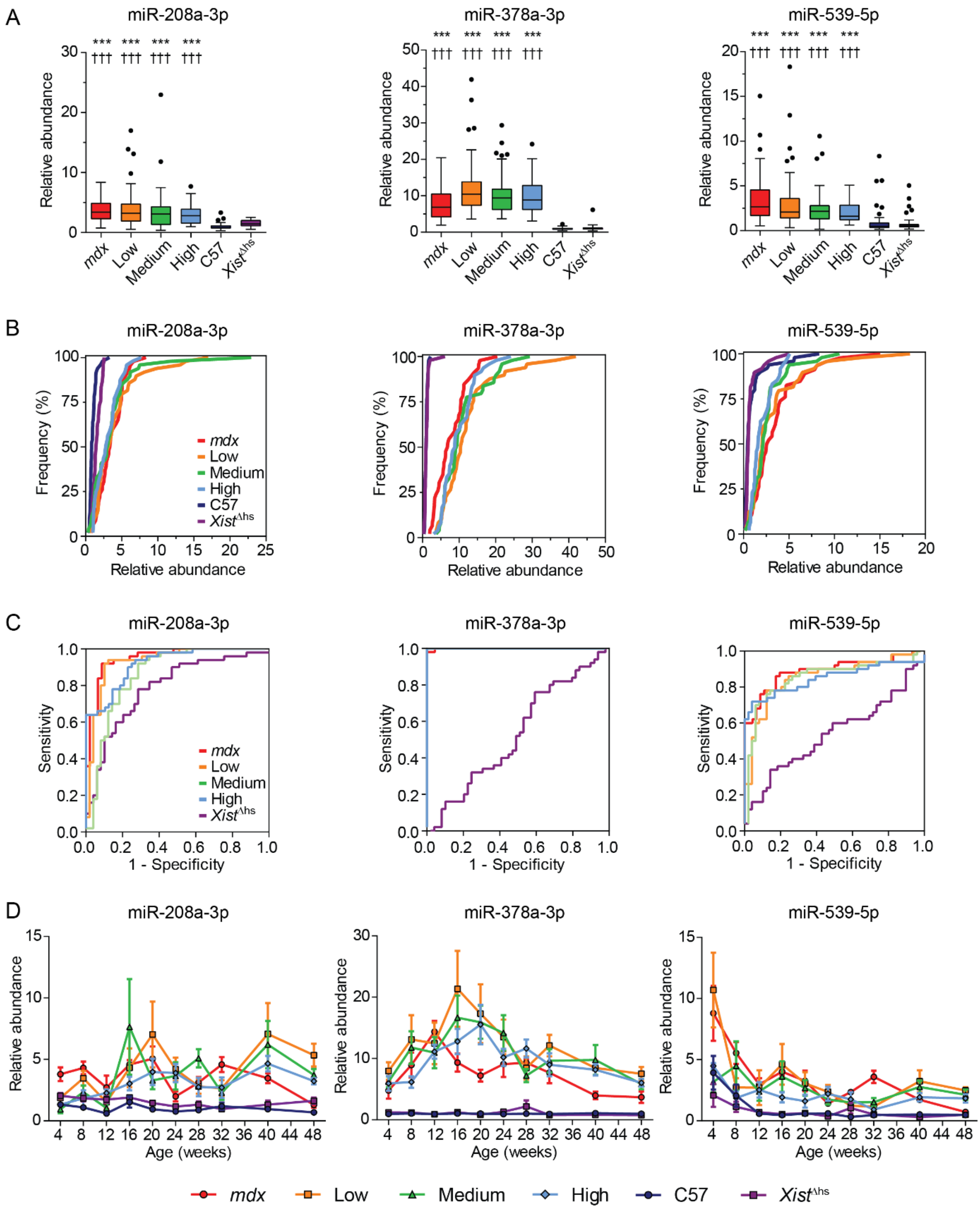
A
miR-208a-3p
miR-378a-3p
miR-539-5p
Relative abundance
mdx
Low
Medium
High
C57
Xist^Δhs
B
Frequency (%)
Relative abundance
C
Sensitivity
1 - Specificity
D
Age (weeks)
mdx
Low
Medium
High
C57
Xist^Δhs

Figure S4

Analysis of individual serum miRNAs: miR-208a-3p, miR-378a-3p and miR-539-5p.

Serum miRNA abundance data for selected individual miRNAs (miR-208a-3p, miR-378a-3p and miR-539-5p) measured using the FirePlex methodology. Data are shown as; (A) Tukey box plots of all measurements pooled for each mouse strain ($n \geq 46$ measurements, range: 46-50), (B) CDF plots comparing experimental groups, (C) ROC curves to assess the potential of each miRNA to distinguish experimental groups from the C57 controls, and (D) time course plots of the mean for every group at each age over the full 4-48 week time period. FirePlex miRNA data were normalized to the geometric average of miR-16-5p, miR-17-5p and miR-92a-3p. Abundance ratios were scaled such that the mean of the C57 control was returned to a value of 1. Values in (D) are mean ± SEM. ***$P<0.001$ for comparisons to the C57 group. †††$P<0.001$ for comparisons to the *Xist*$^{\Delta hs}$ group. *P*-values are for Kruskal-Wallis one-way ANOVA with Dunn's *post hoc* test.

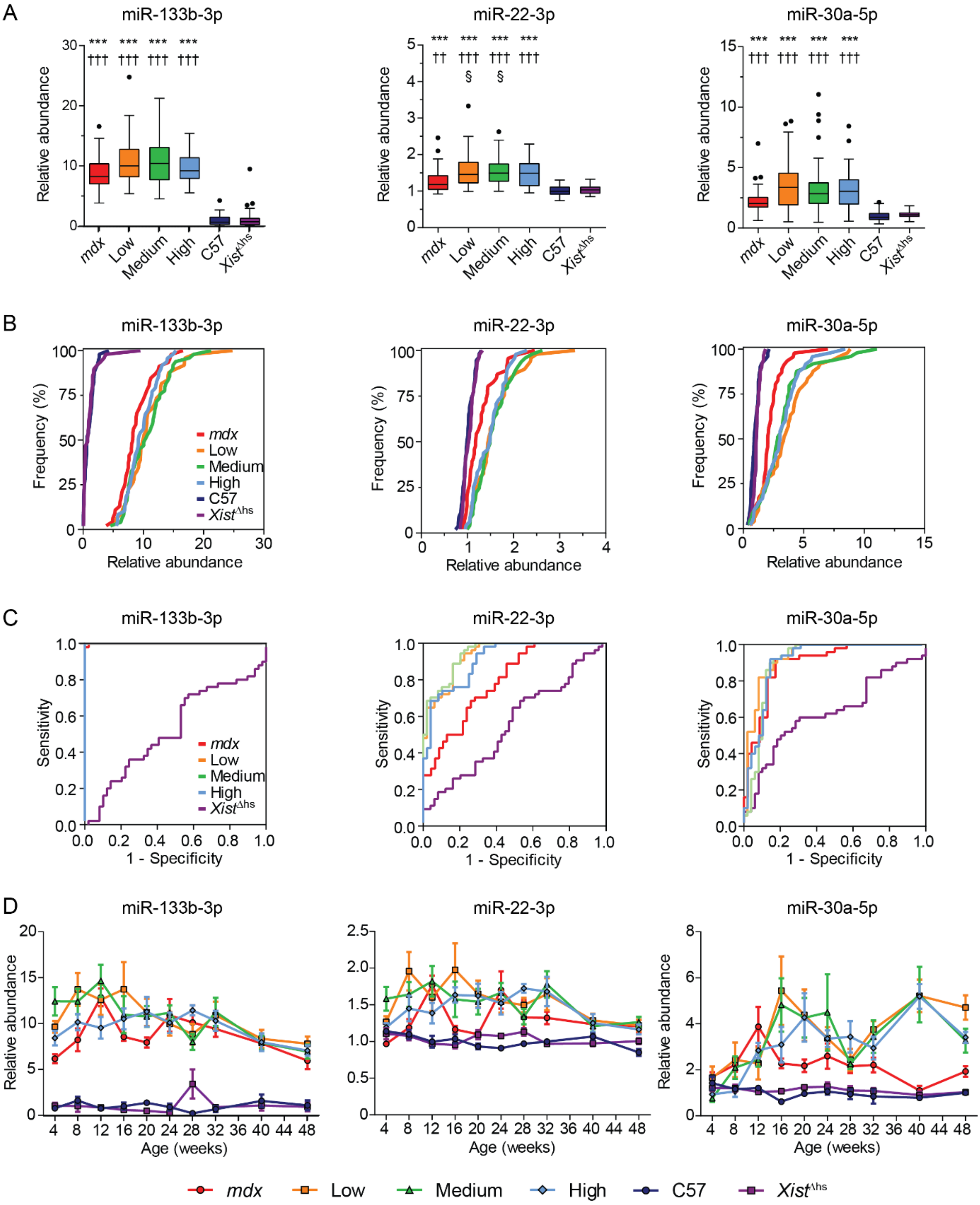
A
miR-133b-3p
miR-22-3p
miR-30a-5p
Relative abundance
***
†††
††
§
mdx
Low
Medium
High
C57
Xist^Δhs
B
Frequency (%)
Relative abundance
C
Sensitivity
1 - Specificity
D
Age (weeks)

Figure S5

Analysis of individual serum miRNAs: miR-133b-3p, miR-22-3p and miR-30a-5p.

Serum miRNA abundance data for selected individual miRNAs (miR-133b-3p, miR-22-3p and miR-30a-5p) measured using the FirePlex methodology. Data are shown as; (A) Tukey box plots of all measurements pooled for each mouse strain ($n \geq 46$ measurements, range: 46-50), (B) CDF plots comparing experimental groups, (C) ROC curves to assess the potential of each miRNA to distinguish experimental groups from the C57 controls, and (D) time course plots of the mean for every group at each age over the full 4-48 week time period. FirePlex miRNA data were normalized to the geometric average of miR-16-5p, miR-17-5p and miR-92a-3p. Abundance ratios were scaled such that the mean of the C57 control was returned to a value of 1. Values in (D) are mean ± SEM. ***$P<0.001$ for comparison with C57 group, †††$P<0.001$ for comparison with the *Xist*$^{\Delta hs}$ group, §$P<0.05$ for comparison to the *mdx* group. *P*-values are for Kruskal-Wallis one-way ANOVA with Dunn's *post hoc* test.

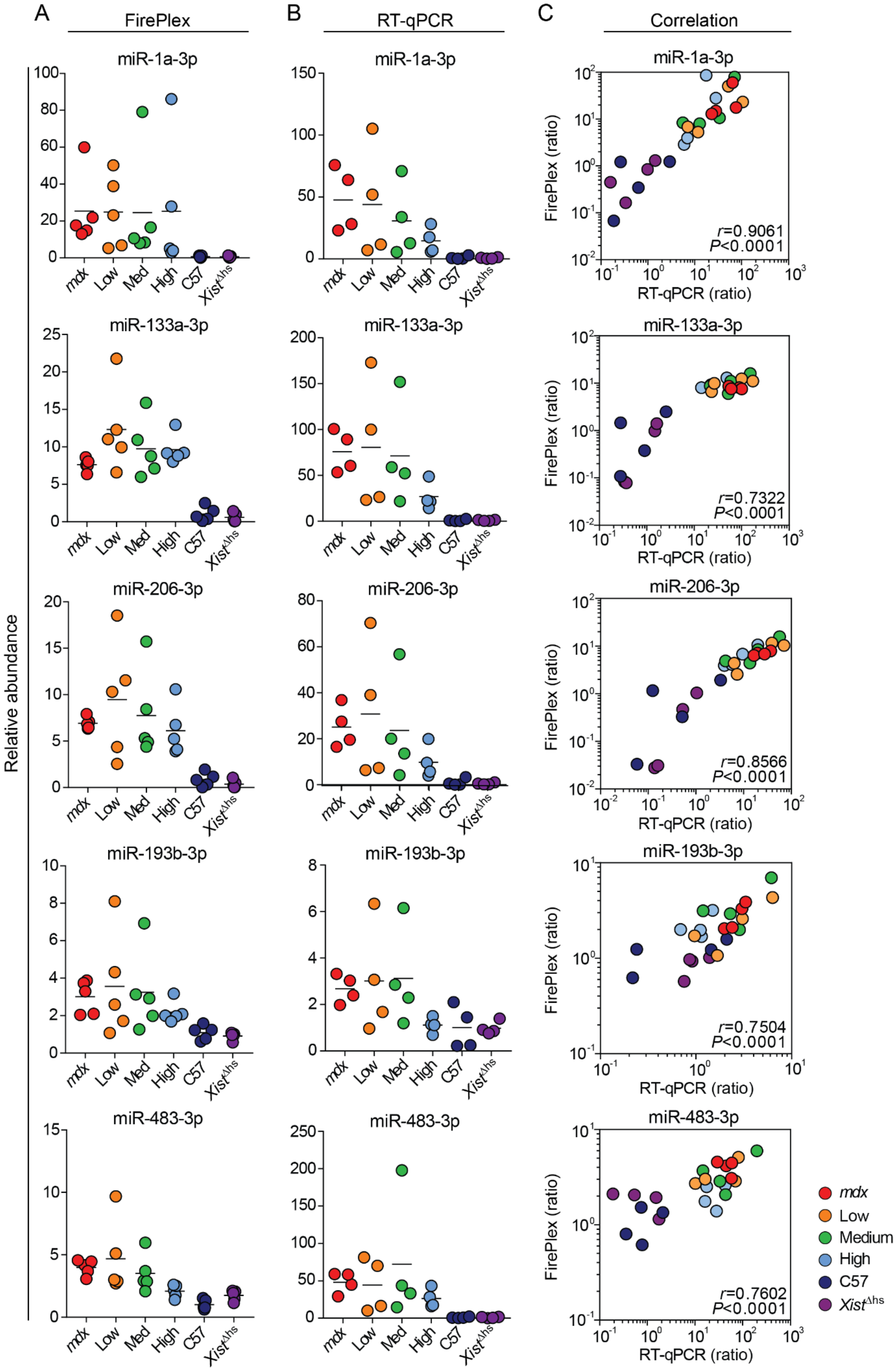

A
FirePlex
B
RT-qPCR
C
Correlation
Relative abundance
miR-1a-3p
miR-133a-3p
miR-206-3p
miR-193b-3p
miR-483-3p
mdx
Low
Med
High
C57
Xist$^{\Delta hs}$
FirePlex (ratio)
RT-qPCR (ratio)
r=0.9061
P<0.0001
r=0.7322
P<0.0001
r=0.8566
P<0.0001
r=0.7504
P<0.0001
r=0.7602
P<0.0001
mdx
Low
Medium
High
C57
Xist$^{\Delta hs}$

Figure S6

RT-qPCR validation of serum miRNA abundance at 16 weeks of age.

Serum samples from 16-week-old mice were reanalyzed by small RNA TaqMan RT-qPCR as orthogonal validation of the FirePlex results. Relative abundance of selected serum miRNAs was visualized for (A) FirePlex data, and (B) RT-qPCR. FirePlex data were normalized to the geometric average of miR-16-5p, miR-17-5p, and miR-92a-3p. RT-qPCR data were normalized to a synthetic spike-in control oligonucleotide (cel-miR-39). The mean value is indicated for each group. (C) Data from the two methodologies were plotted against one another. Pearson's or Spearman's *r* correlation coefficients and correlation *P*-values are shown on the plots as appropriate.

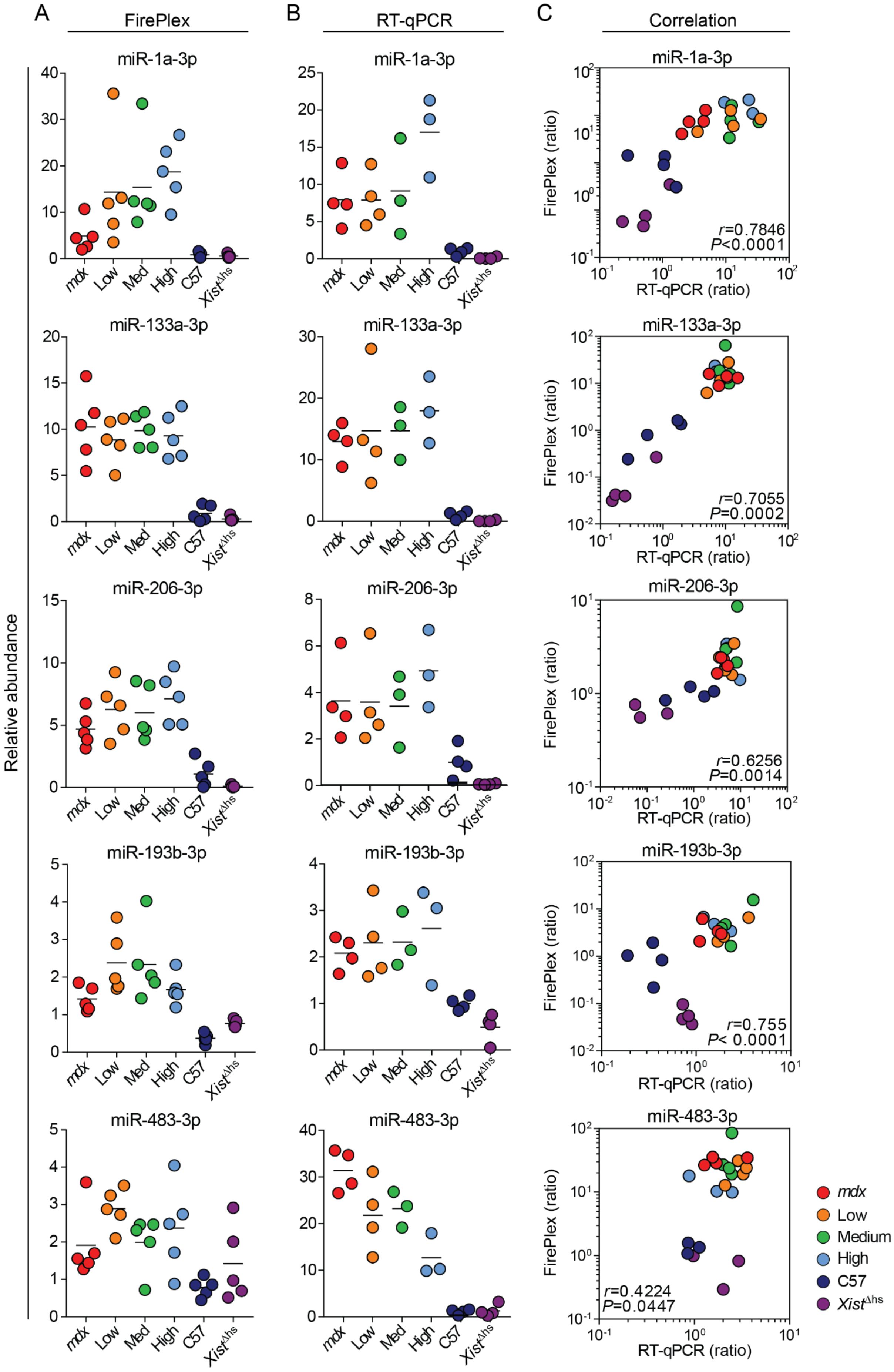

A
FirePlex
B
RT-qPCR
C
Correlation
Relative abundance
miR-1a-3p
miR-133a-3p
miR-206-3p
miR-193b-3p
miR-483-3p
mdx
Low
Med
High
C57
Xist^Δhs
FirePlex (ratio)
RT-qPCR (ratio)
r=0.7846
P<0.0001
r=0.7055
P=0.0002
r=0.6256
P=0.0014
r=0.755
P< 0.0001
r=0.4224
P=0.0447
mdx
Low
Medium
High
C57
Xist^Δhs

Figure S7

RT-qPCR validation of serum miRNA abundance at 24 weeks of age.

Serum samples from 24-week-old mice were reanalyzed by small RNA TaqMan RT-qPCR as orthogonal validation of the FirePlex results. Relative abundance of selected serum miRNAs was visualized for (A) FirePlex data, and (B) RT-qPCR. FirePlex data were normalized to the geometric average of miR-16-5p, miR-17-5p, and miR-92a-3p. RT-qPCR data were normalized to a synthetic spike-in control oligonucleotide (cel-miR-39). The mean value is indicated for each group. (C) Data from the two methodologies were plotted against one another. Pearson's or Spearman's *r* correlation coefficients and correlation *P*-values are shown on the plots as appropriate.

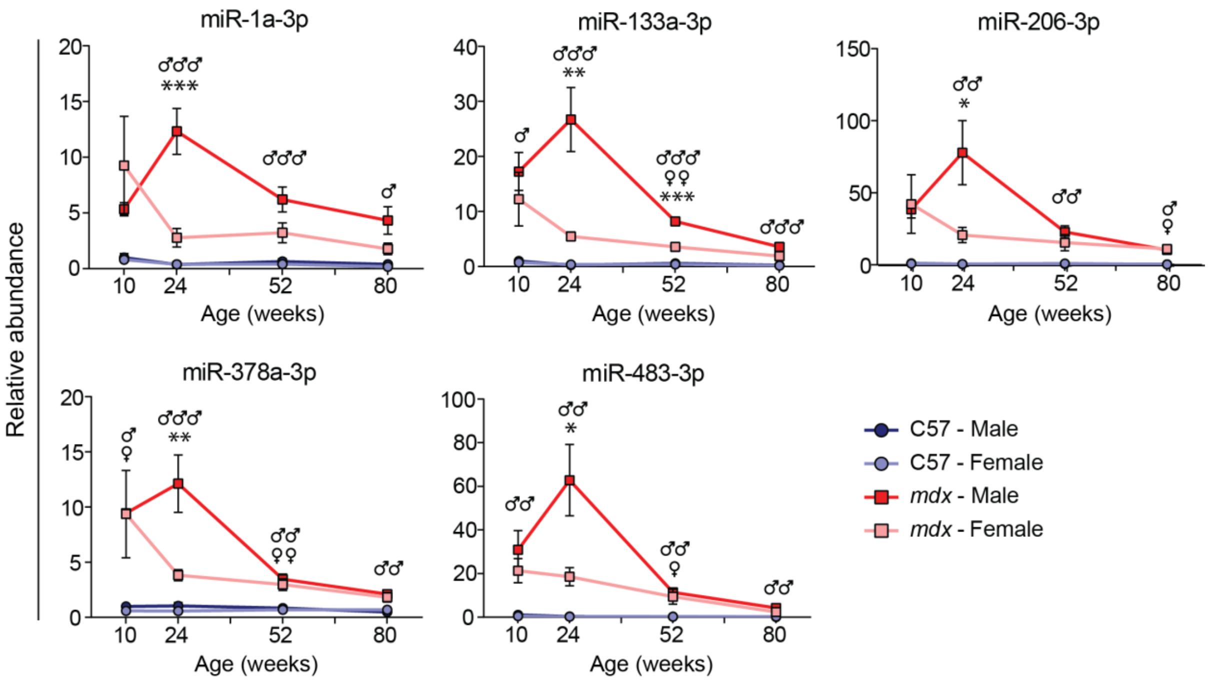

Figure S8

Differential ex-miRNA abundance in dystrophic serum from male and female mice.

Ex-miRNA levels were assessed by small RNA TaqMan RT-qPCR in serum from male and female *mdx* mice at four different ages (10, 24, 52, and 80-weeks-old). Age-matched male and female C57 wild-type mice were used as controls. All groups contained *n*=4 mice except for the 80-week-old C57 females, *n*=3. Abundance ratios were scaled such that the mean of the C57 control was returned to a value of 1. All values are mean ± SEM. ♂*P*<0.05, ♂♂*P*<0.01, ♂♂♂*P*<0.001 comparing male *mdx* with male C57. ♀*P*<0.05, ♀♀*P*<0.01 comparing female *mdx* with female C57. **P*<0.05, ***P*<0.01, ****P*<0.001 comparing male *mdx* with female *mdx*. Statistical significance was assessed by one-way ANOVA at each time point with Tukey *post hoc* test.

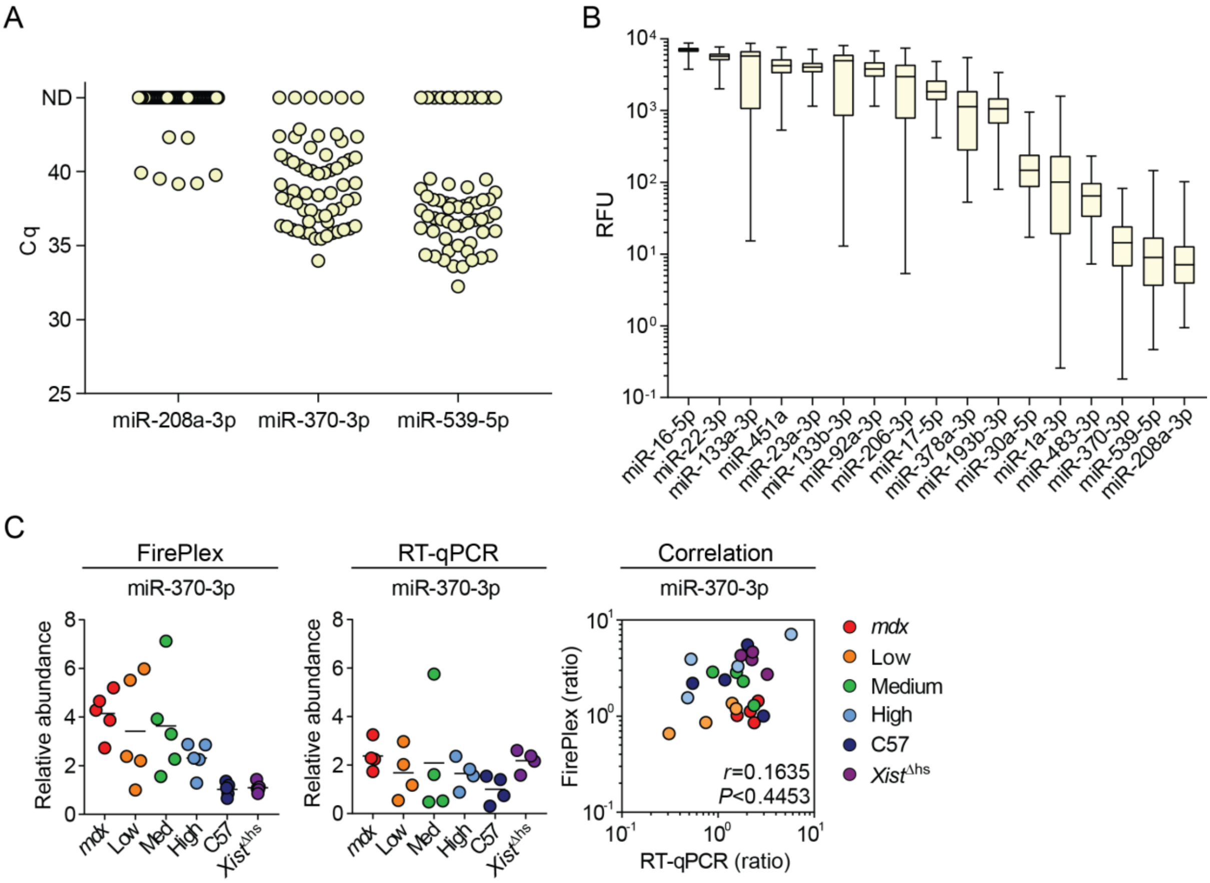

Figure S9

Failure to validate findings for lowly abundant serum miRNAs.

(A) Quantitation cycle (Cq) values for lowly abundant miRNAs as measured by RT-qPCR. ND, not detected. (B) Raw fluorescence units (RFU) for every miRNA analyzed by FirePlex are shown using min/max box plots. (C) Relative, normalized, abundance of 16-week-old serum sample for miR-370-3p as measured by FirePlex and RT-qPCR. FirePlex data were normalized to the geometric average of miR-16-5p, miR-17-5p, and miR-92a-3p. RT-qPCR data were normalized to a synthetic spike-in control oligonucleotide (cel-miR-39). Abundance ratios were scaled such that the mean of the C57 control was returned to a value of 1. The mean values are indicated for each group. Data from the two methodologies were plotted against one another and analyzed by Spearman correlation. The correlation coefficient *r* and *P*-value are shown on the plot.

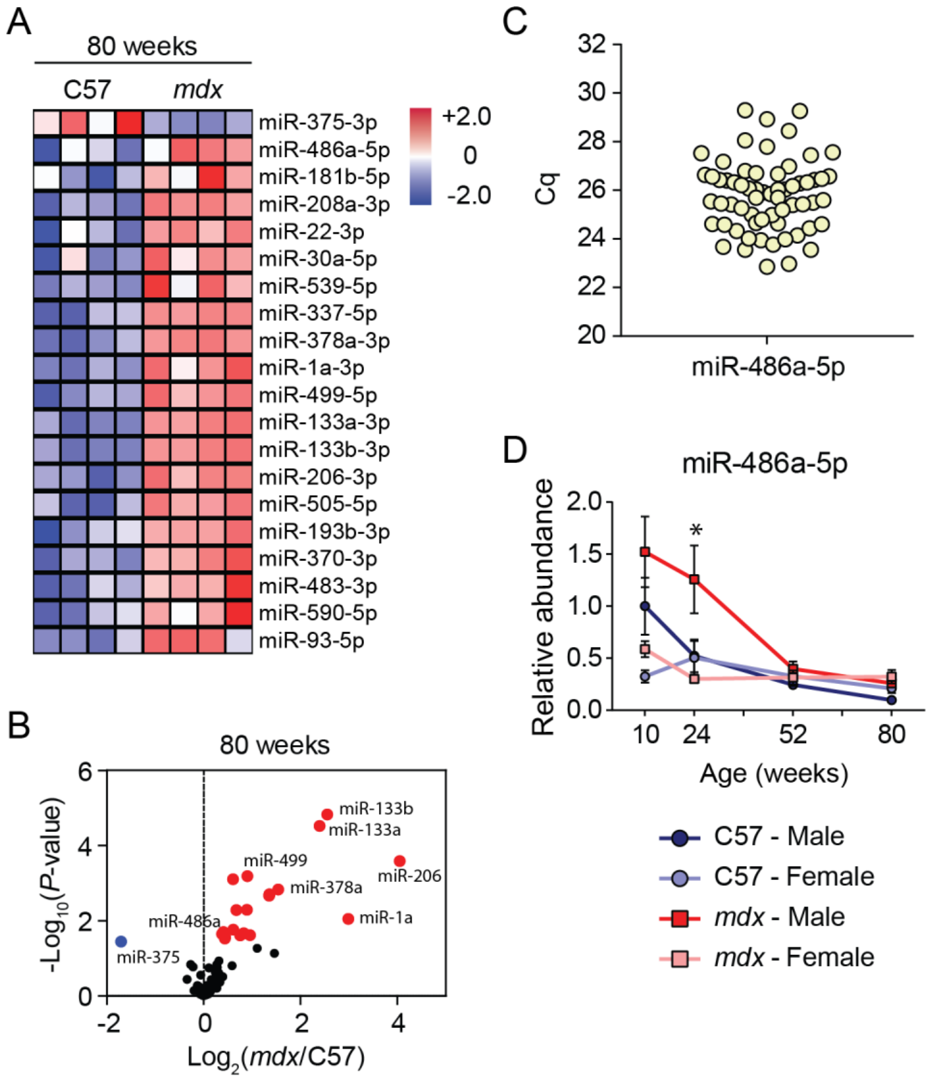
A
80 weeks
C57
mdx
miR-375-3p
miR-486a-5p
miR-181b-5p
miR-208a-3p
miR-22-3p
miR-30a-5p
miR-539-5p
miR-337-5p
miR-378a-3p
miR-1a-3p
miR-499-5p
miR-133a-3p
miR-133b-3p
miR-206-3p
miR-505-5p
miR-193b-3p
miR-370-3p
miR-483-3p
miR-590-5p
miR-93-5p
+2.0
0
-2.0
B
80 weeks
-Log10(P-value)
Log2(mdx/C57)
miR-133b
miR-133a
miR-499
miR-206
miR-378a
miR-486a
miR-1a
miR-375
C
Cq
miR-486a-5p
D
miR-486a-5p
Relative abundance
Age (weeks)
C57 - Male
C57 - Female
mdx - Male
mdx - Female

Figure S10

Profiling of differentially abundant ex-miRNAs in aged dystrophic serum.

The serum of male C57 and *mdx* mice was analyzed using a custom FirePlex miRNA panel (68 miRNAs) at 80 weeks of age. FirePlex miRNA data were normalized to the geometric average of miR-16-5p, miR-17-5p and miR-92a-3p. Differentially abundant miRNAs (P<0.05, Student's t-test) were visualized by (A) heatmap and (B) volcano plot. The scale bar for the heatmap represents row Z-scores with red and blue indicating higher and lower than mean abundance respectively. Statistically significant *mdx* vs C57 changes on the volcano plot are highlighted in red and blue (for elevated and lowered miRNAs respectively). (C) Quantitation cycle (Cq) values for miR-486a-5p as measured by RT-qPCR. (D) Ex-miRNA levels were assessed by small RNA TaqMan RT-qPCR in serum from male and female *mdx* mice at four different ages (10, 24, 52, and 80-weeks-old). Age-matched male and female C57 mice were used as controls. All groups contained n=4 mice except for the 80-week-old C57 females, n=3. RT-qPCR data were normalized to a synthetic spike-in control oligonucleotide (cel-miR-39). All values are mean ± SEM. *P<0.05, comparing male *mdx* with female *mdx*. Statistical significance was assessed by one-way ANOVA at each time point with Tukey *post hoc* test.

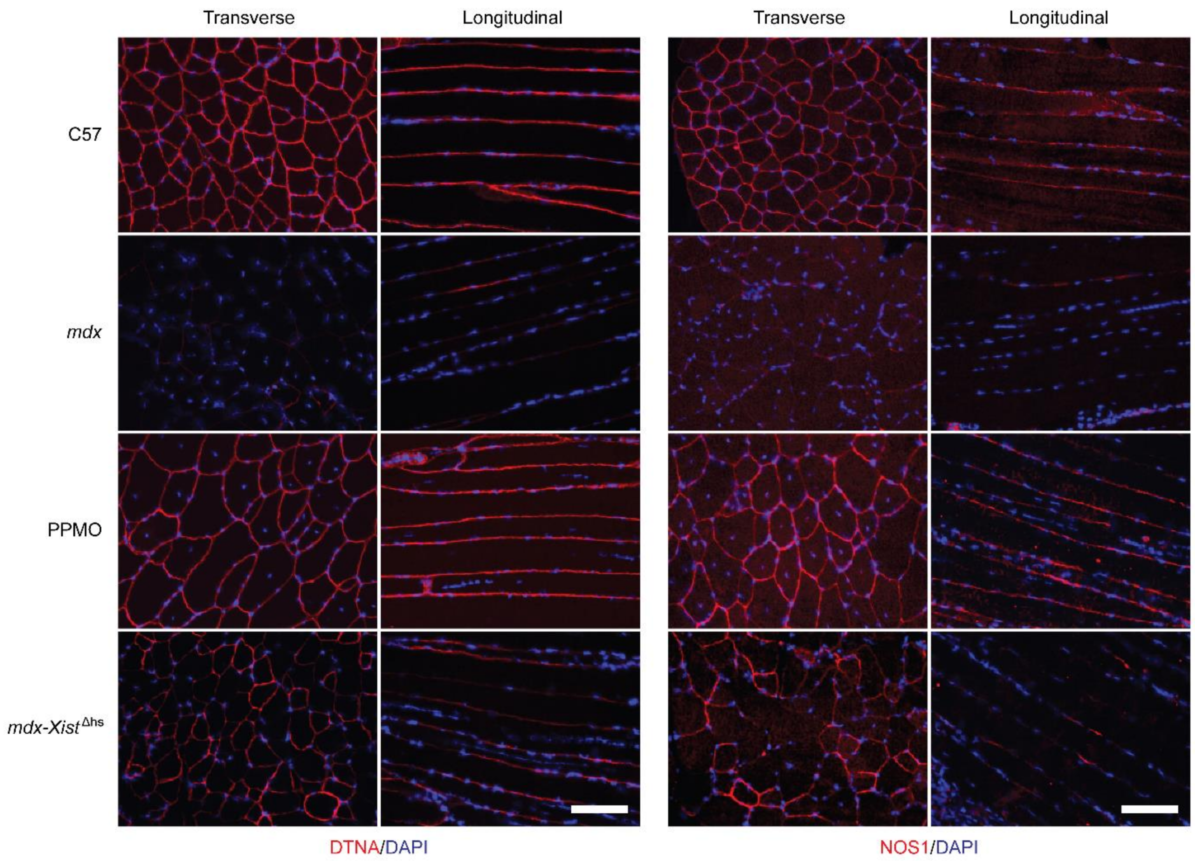

Figure S11

Localization of DTNA and NOS1 expression in PPMO-treated *mdx* and *mdx-Xist*$^{\Delta hs}$ mice.

Representative immunofluorescence staining of dystrobrevin alpha (DTNA) and neuronal nitric oxide synthase (NOS1) in transverse and longitudinal TA sections for C57, *mdx*, PPMO-treated *mdx*, and *mdx-Xist*$^{\Delta hs}$ mice (~40% dystrophin expressing). Sections were co-stained with DAPI to label nuclei. Scale bars represent 100 µm, images taken at 20× magnification.

Supplementary Results and Discussion

Identification of novel candidate biomarkers by serum miRNA profiling

To identify novel putative miRNA biomarkers, serum miRNA profiling was performed at three ages (12, 24, and 48-weeks-old, *n*=4). These ages were selected to represent the progression of dystrophic pathology in the *mdx* mouse, whereby some cardiomyopathic features, such as fibrosis, are observed at 24 weeks and by 48 weeks the dystrophic heart exhibits multiple aberrant cardiac functional parameters [1,2]. miRNA abundance was measured using the FirePlex miRNA assay system and a custom-designed FirePlex panel of 68 miRNA probes which included (a) miRNAs that have previously been identified as elevated in dystrophic serum [3–9], (b) cardiac process-associated miRNAs (cardiomiRs) which are potential markers of dystrophic cardiomyopathy, and (c) putative normalization/quality controls (**Supplementary File S1**).

Differentially abundant miRNAs (*P*<0.05, Student's *t*-test) were visualized by heatmap (**Figure S1A**) and volcano plot (**Figure S1B**) for each age group. Of those miRNAs that were differentially abundant, the majority were found to be increased in *mdx* serum relative to C57 controls. Additionally, the highest number of differentially abundant miRNAs was identified at 12 weeks of age. 14 miRNAs were differentially abundant in *mdx* serum at two or more time points, of which 7 were consistently up-regulated at all three time points (miR-1a-3p, miR-193b-3p, miR-22-3p, miR-30a-5p, miR-370-3p, miR-378a-3p, miR-483-3p; **Figure S1C**). Notably, whereas the myomiR miR-1a-3p was significantly up-regulated at all time points (*P*<0.05), miR-133a/b-3p was differentially expressed at 12 and 24 weeks, and miR-206-3p only at 12 weeks of age. This unexpected finding is likely a consequence of the high variability between replicates at 48 weeks. A total of 12 miRNAs of interest were selected for

further analysis, based on differential abundance at multiple time points and/or previously reported evidence (**Supplementary File S1**).

Quality control analysis

Reference miRNA stability was analyzed using the geNorm method [10] within the Firefly Analysis Workbench and normalization to the geometric average of three miRNAs (miR-16-5p, miR-17-5p and miR-92a-3p) was determined to be the optimal strategy. Variation in non-normalized reference miRNA abundance was minimal between experimental groups (**Figure S2A**, **B**). Correlation analysis of non-normalized, background-subtracted signal intensities illustrated that the reference miRNAs (normalizers) were positively correlated with one another (Spearman *r* range: 0.64 to 0.8, *P*=0) and not correlated with the classical myomiRs (Spearman *r* range: -0.1 to 0.06, not significant) (**Figure S2D**), consistent with a robust normalization strategy. Measurement of the erythrocyte-enriched miRNA, miR-451a, was utilized to assess sample hemolysis [11] as this has the potential to confound ex-miRNA biomarker analysis. miR-451a levels were relatively consistent between samples (**Figure S2C**) and correlated with the abundance of the normalizer control miRNAs (Spearman *r* range: 0.49 to 0.8, *P*=0), indicating that their levels reflect variation in overall miRNA abundance, and that none of the samples were compromised by hemolysis.

Analysis of other putative ex-miRNA biomarkers

miR-208a-3p, miR-378a-3p and miR-539-5p were differentially elevated in dystrophic samples compared to the wild-type controls, but were highly variable over time (**Figure S4A**, **D**). Generally, these miRNAs did not exhibit the same dystrophin-dependent relationship as observed in **Figure 4**, with the exception of miR-539-5p. ROC curve analysis showed that miR-378a-3p exhibited near perfect discrimination between dystrophic and wild-type

samples, but, as with the myomiRs, no correlation with dystrophin expression was observed in *mdx-Xist*$^{\Delta hs}$ mice. In contrast, the cardiomiR, miR-208a-3p, exhibited a lower capacity for distinguishing between healthy and affected mice. Minor, but significant, differences were observed in miR-208a-3p abundance between the C57 and *Xist*$^{\Delta hs}$ control strains. Together, these characteristics make this miRNA less attractive for potential biomarker purposes. Data for the remaining miRNAs (miR-133b-3p, miR-22-3p, and miR-30a-5p) are included for completeness in **Figure S5** and are not discussed further.

Additionally, we did not observe any miRNAs that were positively correlated with age (and by extension disease progression). Instead there appeared to be a global decrease in ex-miRNA abundance with age and consistent with our previous results in DMD patients [9]. This is potentially due to the accumulation of fibrotic tissue in dystrophic muscle which may exert global effects on ex-miRNA release. These results have shown that the relationship between dystrophin protein expression and serum miRNA abundance is miRNA-specific, and highlights the complexity of the miRNA secretome in dystrophin-deficient animals.

Independent validation of profiling results by RT-qPCR

Key findings from the FirePlex analysis were validated using an orthogonal methodology. The totality of the FirePlex data (i.e. aggregated measurements from all time points) clearly showed that there was no dystrophin-dependent effect on ex-myomiR levels in *mdx-Xist*$^{\Delta hs}$ mice. However, when analyzing each time point individually, a dystrophin-dependent relationship was apparent at some time points. This disparity may be explained by technical variability inherent in serum miRNA measurements, or by true biological differences. Inherent biological variability is expected as serum miRNA abundance is a product of release from the entire musculature, with various muscles contributing to different extents depending

on their relative mass and the degree of degenerative/regenerative pathology occurring in a particular muscle at a given time [5]. Therefore, to assess whether the differences observed at different ages are a consequence of technical or biological variation, we selected two time points as representatives of dystrophin-dependent (16 weeks) and dystrophin-independent (24 weeks) relationships, and measured miRNA abundance by RT-qPCR (**Figures S6** and **S7**). The validation data were generally highly consistent with the initial FirePlex results. Some miRNAs (miR-193b-3p and miR-483-3p) exhibited dystrophin-dependent changes in extracellular abundance in *mdx-Xist*$^{\Delta hs}$, but not others (miR-1a-3p, miR-133a-3p and miR-206-3p). Small variations between the FirePlex and RT-qPCR methods were observed for all miRNAs (**Figures S6** and **S7**). However, these are to be expected, especially considering the different data normalization strategies used for each methodology (i.e. geometric average of stable endogenous miRNAs or external spike-in control). Upon closer inspection, individual data points were significantly correlated between the FirePlex and RT-qPCR method for all miRNAs (Spearman r range: 0.43 to 0.96, P<0.0447). Together, these RT-qPCR data confirm the FirePlex results, and suggest that the observed variation in the dystrophin-dependent alterations in ex-myomiR levels over time is a true reflection of biological differences.

Sex-specific variations in ex-miRNA abundance

Given that all the mice used for the FirePlex study were necessarily female (due to the requirement for skewed XCI), we compared serum from male and female *mdx* mice to determine if findings in female mice could be confirmed in the more disease-relevant male model. As such, we performed end-point serum miRNA measurements at 4 ages (10, 24, 52, and 80 weeks) and analyzed both male and female *mdx* and wild-type C57 mice. (The 80 week age time point was included to extend measurements beyond the range of the samples analyzed by FirePlex). Both male and female *mdx* mice exhibited significantly higher ex-

miRNA abundance levels compared with their corresponding C57 controls (**Figure S8**). These findings serve as further validation of the FirePlex data, and also demonstrate the progressive decline in ex-miRNA abundance levels that has been reported in previous studies [9,12,13]. Interestingly, abundance was significantly higher in male compared to female *mdx* mice at 24 weeks of age for all measured miRNAs (P<0.0186). Previous studies have shown that male *mdx* mice exhibit increased sarcolemma permeability at 6 weeks and decreased muscle function at 6 months of age compared to females, highlighting the importance of sex hormone levels in modulating the severity of DMD pathology [14,15], and potentially also in the regulation of ex-miRNA release.

Negative data and inconsistent findings

While the majority of findings from the FirePlex study were independently validated by RT-qPCR (**Figures S6** and **S7**), there were several inconsistencies between measurements for certain miRNAs. We were unable to detect miR-208a-3p, miR-370-3p and miR-539-5p in the majority of the time course samples by RT-qPCR (**Figure S9A**). Inspection of raw FirePlex signal intensities revealed that these miRNAs were very lowly abundant, which likely contributed to the inconsistency in their measurement (**Figure S9B**). miR-370-3p abundance was further analyzed in *mdx*, *mdx-Xist*$^{\Delta hs}$, and control samples at the age of 16 weeks. Correlation between miR-370-3p measurements was poor (Spearman r=0.1635, **Figure S9C**) although the inherent variation in the measurement at Cq values >35 limits the usefulness of this comparison. These differences are most likely attributed to the method of detection, whereby FirePlex measures miRNAs directly from biofluids. In contrast, RT-qPCR requires RNA extraction, certain methods of which are known to exhibit sequence-dependent differences in miRNA extraction efficiency [16,17]. As such, the FirePlex method may exhibit greater sensitivity with respect to the detection of some miRNAs, although the

biological/clinical usefulness of measuring ex-miRNAs with such low abundance levels is debatable.

Considering the progressive nature of the disease, and the age-associated decline in serum abundance levels observed for all ex-miRNA biomarkers investigated thus far, it is important to identify new biomarkers that either stay consistent over time, or are correlated with disease progression. For this purpose, we performed an additional FirePlex screening at 80 weeks of age in male C57 and *mdx* mice with the panel of 68 miRNAs described above (**Supplementary File S1**). Of the 19 up-regulated miRNAs in the *mdx* mice (Student's *t*-test, P<0.05, **Figure S10A**,**B**), we selected miR-486a-5p for further investigation because it was only called as being differentially abundant 80 weeks of age, making it a potential marker for late-stage disease progression. miR-486a-5p was observed to be relatively highly abundant in serum (mean Cq = 25.8), although neither male or female *mdx* samples were significantly different from their respective controls when measured by RT-qPCR (**Figure S10C**,**D**). Furthermore, miR-486a-5p also exhibited a progressive decrease in miR-486a-5p abundance over time, suggesting that this miRNA offers no advantage over existing (and arguably more promising) biomarker candidates. While ex-miRNAs may have use for monitoring disease progression and/or response to therapy in younger patients, it may prove difficult to identify a serum miRNA biomarker that increases over time with disease progression, possibly as a consequence of a general impairment in miRNA release from aged dystrophic muscle.

Supplementary References

[1] Stuckey DJ, Carr CA, Camelliti P, Tyler DJ, Davies KE, Clarke K. In vivo MRI characterization of progressive cardiac dysfunction in the mdx mouse model of muscular dystrophy. PLoS ONE 2012;7:e28569. doi:10.1371/journal.pone.0028569.

[2] Van Erp C, Loch D, Laws N, Trebbin A, Hoey AJ. Timeline of cardiac dystrophy in 3-18-month-old MDX mice. Muscle Nerve 2010;42:504–13. doi:10.1002/mus.21716.

[3] Cacchiarelli D, Legnini I, Martone J, Cazzella V, D'Amico A, Bertini E, et al. miRNAs as serum biomarkers for Duchenne muscular dystrophy. EMBO Mol Med 2011;3:258–65. doi:10.1002/emmm.201100133.

[4] Mizuno H, Nakamura A, Aoki Y, Ito N, Kishi S, Yamamoto K, et al. Identification of muscle-specific microRNAs in serum of muscular dystrophy animal models: promising novel blood-based markers for muscular dystrophy. PLoS ONE 2011;6:e18388. doi:10.1371/journal.pone.0018388.

[5] Roberts TC, Blomberg KEM, McClorey G, Andaloussi SE, Godfrey C, Betts C, et al. Expression Analysis in Multiple Muscle Groups and Serum Reveals Complexity in the MicroRNA Transcriptome of the mdx Mouse with Implications for Therapy. Molecular Therapy — Nucleic Acids 2012;1:e39. doi:10.1038/mtna.2012.26.

[6] Roberts TC, Godfrey C, McClorey G, Vader P, Briggs D, Gardiner C, et al. Extracellular microRNAs are dynamic non-vesicular biomarkers of muscle turnover. Nucl Acids Res 2013;41:9500–13. doi:10.1093/nar/gkt724.

[7] Vignier N, Amor F, Fogel P, Duvallet A, Poupiot J, Charrier S, et al. Distinctive serum miRNA profile in mouse models of striated muscular pathologies. PLoS ONE 2013;8:e55281. doi:10.1371/journal.pone.0055281.

[8] Jeanson-Leh L, Lameth J, Krimi S, Buisset J, Amor F, Le Guiner C, et al. Serum profiling identifies novel muscle miRNA and cardiomyopathy-related miRNA biomarkers in Golden Retriever muscular dystrophy dogs and Duchenne muscular dystrophy patients. Am J Pathol 2014;184:2885–98. doi:10.1016/j.ajpath.2014.07.021.

[9] Coenen-Stass AML, Sork H, Gatto S, Godfrey C, Bhomra A, Krjutškov K, et al. Comprehensive RNA-Sequencing Analysis in Serum and Muscle Reveals Novel Small RNA Signatures with Biomarker Potential for DMD. Mol Ther Nucleic Acids 2018;13:1–15. doi:10.1016/j.omtn.2018.08.005.

[10] Vandesompele J, Preter KD, Pattyn F, Poppe B, Roy NV, Paepe AD, et al. Accurate normalization of real-time quantitative RT-PCR data by geometric averaging of multiple internal control genes. Genome Biology 2002;3:research0034. doi:10.1186/gb-2002-3-7-research0034.

[11] Blondal T, Jensby Nielsen S, Baker A, Andreasen D, Mouritzen P, Wrang Teilum M, et al. Assessing sample and miRNA profile quality in serum and plasma or other biofluids. Methods 2013;59:S1-6. doi:10.1016/j.ymeth.2012.09.015.

[12] Coenen-Stass AML, Betts CA, Lee YF, Mäger I, Turunen MP, El Andaloussi S, et al. Selective release of muscle-specific, extracellular microRNAs during myogenic differentiation. Hum Mol Genet 2016;25:3960–74. doi:10.1093/hmg/ddw237.

[13] Zaharieva IT, Calissano M, Scoto M, Preston M, Cirak S, Feng L, et al. Dystromirs as serum biomarkers for monitoring the disease severity in duchenne muscular dystrophy. PLoS ONE 2013;8:e80263. doi:10.1371/journal.pone.0080263.

[14] Hourdé C, Joanne P, Noirez P, Agbulut O, Butler-Browne G, Ferry A. Protective effect of female gender-related factors on muscle force-generating capacity and fragility in the dystrophic mdx mouse. Muscle Nerve 2013;48:68–75. doi:10.1002/mus.23700.

[15] Salimena MC, Lagrota-Candido J, Quírico-Santos T. Gender dimorphism influences extracellular matrix expression and regeneration of muscular tissue in mdx dystrophic mice. Histochem Cell Biol 2004;122:435–44. doi:10.1007/s00418-004-0707-8.
[16] Monleau M, Bonnel S, Gostan T, Blanchard D, Courgnaud V, Lecellier C-H. Comparison of different extraction techniques to profile microRNAs from human sera and peripheral blood mononuclear cells. BMC Genomics 2014;15. doi:10.1186/1471-2164-15-395.
[17] Brown RAM, Epis MR, Horsham JL, Kabir TD, Richardson KL, Leedman PJ. Total RNA extraction from tissues for microRNA and target gene expression analysis: not all kits are created equal. BMC Biotechnol 2018;18:16. doi:10.1186/s12896-018-0421-6.
